# Physiological Mechanisms and Life History Trade‐Offs in Salmonids Shape In‐Tissue Correlations of an Essential Micronutrient

**DOI:** 10.1002/ece3.72339

**Published:** 2025-10-16

**Authors:** Maciej Jan Ejsmond, Vittoria Todisco, Marc M. Hauber, Kjetil Hindar, Samuel Hylander

**Affiliations:** ^1^ Department of Biosciences, Faculty of Mathematics and Natural Sciences University of Oslo Oslo Norway; ^2^ Centre for Ecology of Microbial Model Systems Linnaeus University Kalmar Sweden; ^3^ Institute of Environmental Sciences Jagiellonian University Krakow Poland; ^4^ Norwegian Institute for Nature Research Trondheim Norway

**Keywords:** fish, life‐history, reproduction, salmon, thiamine, vitamin

## Abstract

The lack of a fitness‐based theory of micronutrient allocation to specific tissues hinders understanding of the ultimate causes of mass juvenile mortality due to thiamine (vitamin B1) deficiency, which is an emerging threat to marine and coastal ecosystems worldwide. We modeled the optimal allocation of thiamine in salmon to somatic and reproductive tissues to investigate correlations between tissue thiamine levels, adult mortality, juvenile recruitment, and excretion rates that change with thiamine concentration. The model showed a positive correlation between thiamine levels in gonads and muscles, with a slope that increased with time. This was driven by a constrained thiamine input in salmon, but a negative or no correlation was found in scenarios with high thiamine input. These predictions were confirmed by analysis of empirical data from Atlantic salmon ( *Salmo salar* ) populations that differ in the occurrence of episodic thiamine deficiency. A positive correlation was indicative of low thiamine input, regardless of how juvenile recruitment and adult survival increased with thiamine concentration. The model output suggests that renal (i.e., kidney) reuptake is fundamental to understanding micronutrient allocation strategies. Measuring correlations between micronutrient concentrations in reproductive and somatic tissues of adults may help to detect early signs of thiamine deficiency before mass mortality of juveniles occurs. This can complement the previously suggested tissue concentrations and food web indicators. Future studies should try to distinguish and quantify the factors that alter the net thiamine input in salmonids and the subsequent allocation to offspring. Particular attention should be given to changes in thiamine uptake from the diet, including intestinal uptake mechanisms and effects of thiaminase activity. Additionally, more information is needed on internal factors that reduce thiamine availability, such as thiamine degradation as an antioxidant during lipid metabolism, and other physiological factors that can potentially increase thiamine loss, including allocation mechanisms and renal processes.

## Graphical abstract

The lack of a fitness‐based theory for micronutrient allocation hinders understanding of juvenile mortality due to thiamine deficiency, a threat to organisms in aquatic ecosystems. Our model showed that thiamine levels in gonads and muscles correlate positively, indicating low thiamine input, and this was confirmed by empirical data from Atlantic salmon. Renal reuptake is crucial for understanding micronutrient strategies, and measuring tissue correlations can detect early thiamine deficiency signs.

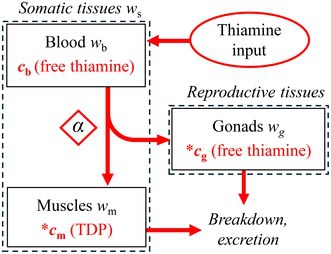

## 1 Introduction

Spatiotemporal variation in the composition of available dietary nutrients is one of the most important drivers of the evolution of life history traits and developmental patterns (e.g., Filipiak and Weiner 2014; O'Brien et al. 2004; Swanson et al. 2016). While the evolution of life histories and foraging behavior is primarily considered in the context of the dietary composition of macronutrients, for example, nitrogen, phosphorus, and sodium (e.g., Kaspari 2020; O'Brien et al. 2004; Snell‐Rood et al. 2015), little attention has been paid to constraints imposed by vitamin availability. These key micronutrients comprise a diverse group of chemicals that are essential for proper physiological functioning, are required in small amounts, and must be obtained through the diet, with some degree of provisioning from the gut microbiota (Patel 2020). Among the diverse chemical group of vitamins, B vitamins share the property of being key precursors and coenzymes essential for the metabolism of amino acids, sugars, fatty acids, and nucleic acids (Combs 2012; Hrubša et al. 2022; Patel 2020). While the role of B vitamins in the proper functioning of numerous physiological processes in metazoans and in the control of oxidative stress is well established (Hrubša et al. 2022; Patel 2020; van de Lagemaat et al. 2019), their role in structuring ecosystems is a subject of intense debate, fueled by the vast ecological impact of episodic deficiencies of these vitamins in consumers (Gilbert 2018; Harder et al. 2018; Hylander et al. 2024; Kraft and Angert 2017; Majaneva et al. 2020; Wang et al. 2023). In particular, the deficiency of thiamine (vitamin B_1_), a flagship B vitamin, and its relationship to population declines has been identified as one of the most important conservation issues in coastal marine ecosystems in the Northern Hemisphere (Gilbert 2018; Sutherland et al. 2018). Thiamine is multifunctional, most notably as a cofactor in several central metabolic pathways, for example, in the pentose phosphate pathway and Krebs cycle (Combs 2012). It occurs in different forms where thiamine pyrophosphate (TDP) is the metabolically active constituent and a phosphorylated form of free thiamine (Combs 2012). In recent decades, several species of top consumers from diverse taxonomic groups, including piscivorous fish and seabirds, have experienced episodic events of mass juvenile mortality associated with lethal and sublethal thiamine levels and characteristic symptoms of paralysis (Balk et al. 2016, 2009; Fitzsimons et al. 1999; Mikkonen et al. 2011; Mörner et al. 2017). This phenomenon affects several coastal ecosystems in the Northern Hemisphere, including the Baltic Sea, the Great Lakes, New York Finger Lakes, and California's Central Valley (Balk et al. 2016; Bengtsson et al. 1999; Fitzsimons et al. 1999; Harder et al. 2018; Majaneva et al. 2020; Mantua et al. 2025; Mantua et al. 2021; Reed et al. 2023).

The episodes of ‘early mortality’ are often drastic in magnitude and have been suggested to be capable of creating demographic cohort gaps in populations originating from certain spawning sites (Harder et al. 2018; Karlström 1999). In years of thiamine deficiency‐induced early mortality, females are able to produce offspring in terms of biomass but fail to supply sufficient thiamine reserves that would ensure proper physiological functioning in the first days of life and successful recruitment thereafter (Amcoff et al. 1998; Lundström et al. 1998). This raises the question of if and how a female's allocation of the lacking micronutrient between somatic and reproductive tissues consequently affects fitness. The strategy of allocating thiamine between somatic and reproductive tissues represents the classic trade‐off between current and future reproduction (cf. Williams 1966); in the case of a limited micronutrient, a female faces an evolutionary dilemma of whether to prioritize her own survival by allocating the vitamin to somatic tissues or to ensure offspring recruitment by allocating the scarce micronutrient to reproductive tissues.

The micronutrient balance of an organism depends on the levels of input and excretion. There are several hypotheses suggesting that thiamine deficiency occurs in top consumers as a result of disruption of thiamine input due to lower transfer in the food web, increased presence of enzymes (e.g., thiaminase) that degrade thiamine, or elevated thiamine depletion when the vitamin functions as a site‐specific antioxidant against lipid peroxidation (Ejsmond et al. 2019; Fitzsimons et al. 1999; Fridolfsson et al. 2020; Harder et al. 2018; Hylander et al. 2024; Keinänen et al. 2012; Rasmussen et al. 2023; Sannino et al. 2018; Vuorinen et al. 2020). It is unclear which of these mechanisms predominates and to what extent they contribute to the episodic mass mortality induced by thiamine deficiency.

Thiamine concentrations in common prey items of salmonids are generally regarded to be sufficient to meet the demands of the fish (Keinänen et al. 2012; Woodward 1994), suggesting that disruptions of the food web in terms of thiamine production would be of less importance for top consumers. The thiaminase and lipid peroxidation hypotheses have been extensively studied (e.g., Harder et al. 2018; Keinänen et al. 2012; Vuorinen et al. 2021; Vuorinen et al. 2020). The occurrence of species exhibiting thiaminase activity as well as lipid‐rich diets, especially in polyunsaturated fatty acids, has both been correlated with the outbreaks of thiamine deficiency (Harder et al. 2018; Keinänen et al. 2012). Thiaminase has been suggested to degrade thiamine in the gastrointestinal tract before uptake in the fish (Harder et al. 2018; Honeyfield et al. 2005; Houde et al. 2015). On the other hand, lipid‐rich diets have instead been suggested to expose top consumers to a depletion of thiamine if it is used as a site‐specific antioxidant to avoid lipid peroxidation during metabolism (Keinänen et al. 2012). There are examples of negative correlations between fish lipid concentration and free thiamine in muscle supporting this hypothesis (Keinanen et al. 2022). However, fish exhibiting thiaminase activity are generally lipid‐rich (Rowland et al. 2023), confounding the possibility of distinguishing between potential thiaminase or lipid effects. While considerable attention has been paid to disruptions in the thiamine input and loss processes described above (i.e., thiaminase and loss due to antioxidant activity), little is known about the potential role of physiological mechanisms of thiamine excretion in maintaining micronutrient balance under chronic disruptions of the input (Koski et al. 2005, 2001). Thiamine is a water‐soluble organic cation, and renal excretion is the major route of thiamine loss (Gastaldi et al. 2000; Hrubša et al. 2022). It would be difficult for a bird, mammal, or fish to maintain micronutrient homeostasis without mechanisms of active transport that allow for both reabsorption and excretion (Beyenbach 2004). Thiamine is a water‐soluble micronutrient readily filtered by renal glomeruli, reabsorbed at low concentrations into the renal venous circulation via high‐affinity active transporters in the renal brush border membrane (Gastaldi et al. 2000; Hrubša et al. 2022). Thiamine reabsorption switches to active secretion when concentrations of the micronutrient are high (Gastaldi et al. 2000; Hrubša et al. 2022), suggesting also the potential physiological ceiling for clearing of plasma thiamine levels with active secretion. The mechanisms of renal reuptake and active excretion make physiological elimination rates strongly dependent on overall thiamine concentration in the tissues. Thus, considerations of an optimal strategy of thiamine allocation to different tissues must be made in concert with the physiological processes of thiamine excretion, as changes in concentration would alter not only fitness payoffs but also rates of thiamine loss. To our knowledge, such an interaction between life history trade‐offs and physiological mechanisms of micronutrient excretion has not been investigated in the life history evolution literature.

Thiamine deficiency has been identified in several studies as the proximate cause of episodic mass mortality of yolk‐sac fry and birds (e.g., Balk et al. 2016; Balk et al. 2009; Bengtsson et al. 1999; Fitzsimons et al. 1999; Harder et al. 2018; Mörner et al. 2017) although the deficiency in birds has been disputed (Sonne et al. 2012; Tillitt et al. 2012). There is an urgent need for measures that would help detect early signs of thiamine deficiency long before it takes effect during the spawning season, but early detection of this ecological threat is problematic. In salmonids in which thiamine deficiency has been widely documented, the severity of early mortality can vary widely, with recruitment reaching healthy population levels in some years and nearly all juveniles dying within days of spawning in other years (e.g., Majaneva et al. (2020); Vuorinen et al. (2021)). While female thiamine status can provide some information, it is also highly variable within populations and along the life cycle (Todisco, Fridolfsson, et al. 2024; Todisco, Hauber, et al. 2024). To maximize fitness, a female constrained by thiamine input would be expected to modify the strategy of allocation of the lacking micronutrient to somatic and reproductive tissues to optimize adult survival, offspring recruitment, and to reduce thiamine losses due to physiological processes of excretion. Such a change in the strategy of managing micronutrient balance may be reflected in differences in tissue thiamine concentrations between populations with high and low thiamine input. In turn, it would provide a valuable indicator of the coming mass mortality episode. Importantly, correlations between thiamine tissue levels may serve as a complementary measure to female thiamine status and help in the early detection of ecosystems vulnerable to negative effects of thiamine deficiency, both in salmon and in species that are less studied lacking baseline data on thiamine deficiency threshold concentrations. This can complement the more traditional way of detecting thiamine deficiency with concentrations of thiamine in liver and eggs along with food web indicators such as correlations between thiamine deficiency and the abundance of certain planktivorous fish (Keinänen et al. 2024; Keinänen et al. 2012; Majaneva et al. 2020; Mikkonen et al. 2011; Vuorinen et al. 2024; Vuorinen et al. 2021).

In this work, we present a life history model of a female fish in which allocation of thiamine to locomotor (muscles) and reproductive tissues (gonads) affected adult survival and juvenile recruitment, respectively. Model females were exposed to different levels of thiamine input. Females optimized fitness by allocating available thiamine to muscles and gonads, with rates of vitamin loss from the body being determined by processes of renal reuptake at low thiamine levels and active secretion at high thiamine levels. The model shows how the input level of thiamine translates into correlations between tissue concentration in muscles and gonads, and the role of life history trade‐offs and excretion in shaping these correlations. The theoretical predictions were compared with empirical data on thiamine levels in female Atlantic salmon ( *Salmo salar* ) sampled at different stages of the spawning season in two systems, the North Atlantic Ocean and the Baltic Sea, that differ in the occurrence of thiamine deficiency episodes (Todisco, Fridolfsson, et al. 2024; Todisco, Hauber, et al. 2024). Our model considers the trade‐off between current and future reproduction, manifested by thiamine level–dependent adult survival and juvenile recruitment, and the physiological mechanisms of micronutrient excretion. By combining elements of physiology and life history evolution, our work contributes to the development of a general theory of optimal micronutrient allocation.

## 2 Materials and Methods

### 2.1 The Model

#### 2.1.1 Outline

**FIGURE 1:**
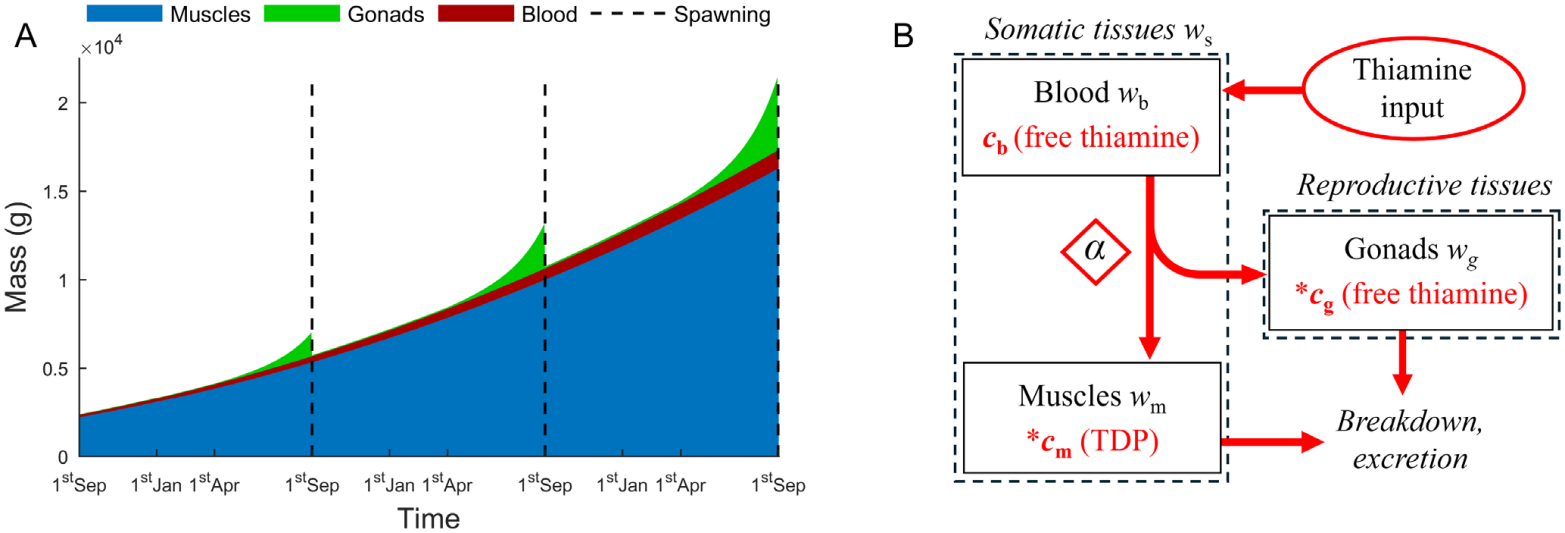
Temporal variation in tissue mass and a scheme of the micronutrient allocation model. (A) Changes in mass of somatic tissues and gonads (see legend) illustrate life of a female of Atlantic salmon. (B) The graph of thiamine flow (red arrows) between modeled somatic and reproductive tissues (outlined with dashed lines). The thiamine input scenarios determine the blood level *c*_b_. The strategy of allocation of the vitamin from the blood between gonads and muscles (outlined with solid lines) is set by the allocation strategy *α* (red diamond), which takes the value from 0 to 1 at each day of life, and divides the pool of free thiamine in the blood between gonads and muscles. Free thiamine allocated to the muscles is converted into the active thiamine form TDP. The rate of thiamine loss from muscles and gonads depends on concentration of the vitamin in these tissues. The strategy α evolves to maximize fitness and depends on the concentration of free thiamine in the gonads *c*_g_ and TDP level in the muscles *c*_m_ (asterisks).

**FIGURE 2:**
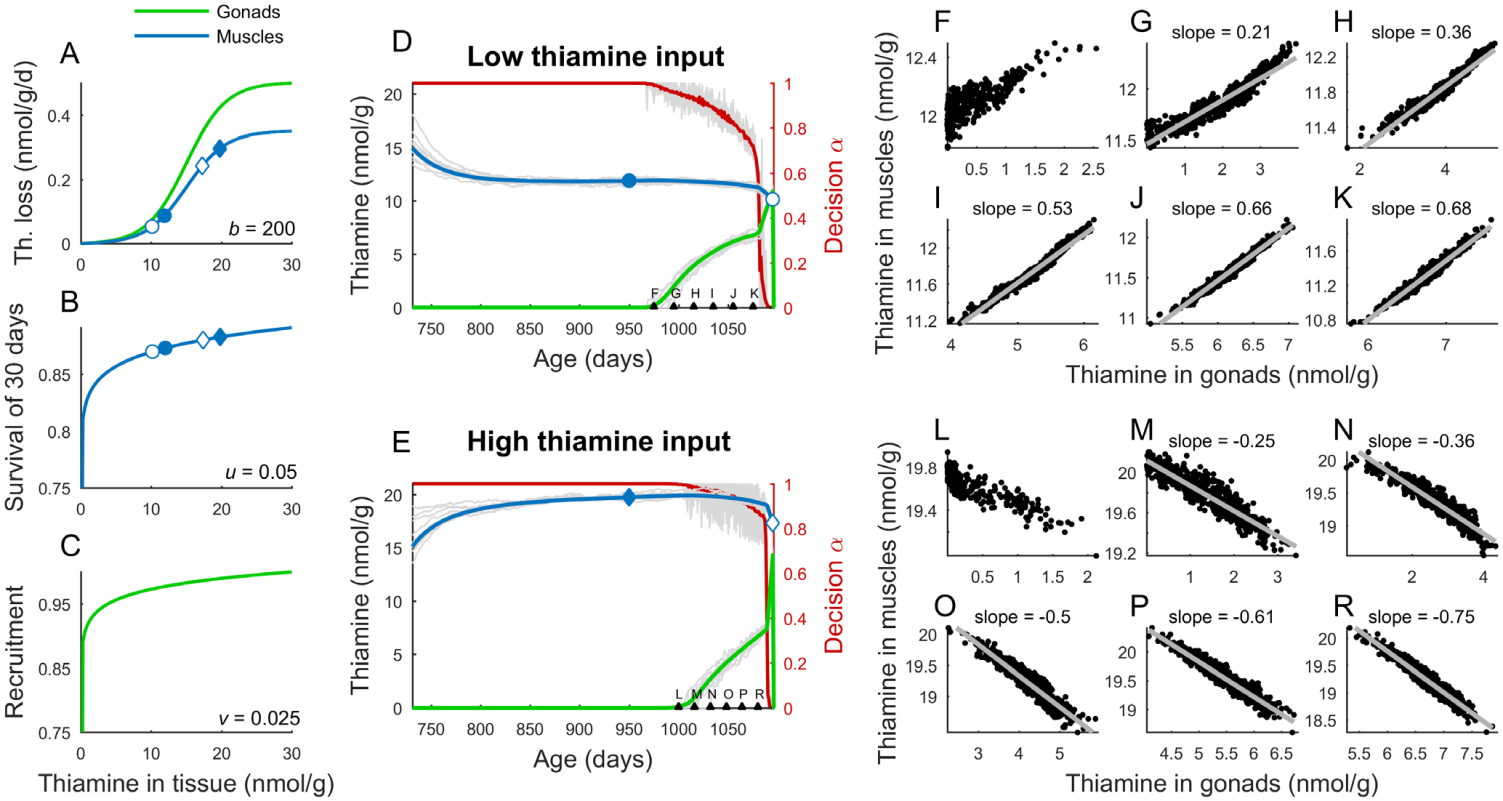
Key model assumptions and the effect of thiamine input. (A–C) The rate of thiamine loss, adult survival, and offspring recruitment depended on thiamine concentration in muscles and gonads. (D, E) The dynamics of thiamine concertation in gonads and muscles for scenarios of low and high thiamine input with thiamine blood level *c*_b_ of 2 and 6 nmol/g respectively. Changes in thiamine tissue levels are presented along with the proportion of blood thiamine allocated between muscles (*α* = 1 corresponds to all thiamine converted to TDP in muscles) and gonads (*α* = 0 corresponds to all thiamine allocated to gonads). Circles and diamonds in D–E, match specific thiamine levels that correspond to rates of thiamine loss, adult survival, and juvenile recruitment depicted by circles and diamonds in A–C. The individual variation is represented by the light gray lines. Points labeled F–R match days for calculated linear regressions shown in panels F–R. (F–R) Linear regressions, with slopes given at the top of panels, for (F–K) low and (L–R) high thiamine input scenarios. (A–C) Scenarios were modeled with the values of the exponents *b*, *u* and *v* (cf. Equations (1), (2), and (4)), given in the lower right corner of the panels.

The model examines the strategy of thiamine allocation between muscles and gonads by a female fish. The temporal changes in the mass of tissues and the timing of reproduction were parameterized to resemble the life history of an iteroparous marine top consumer, Atlantic salmon (Figure 1A). However, some salmonids, such as Pacific salmon, for example, coho salmon ( *Oncorhynchus kisutch* ) and chinook salmon ( *O. tshawytscha* ) (Crespi and Teo 2002), are semelparous, and we modeled semelparous life histories as well (see Figure S1). We simulated different scenarios of thiamine availability approximated by the level of thiamine input (Figure 1B). For simplicity, we did not consider whether thiamine input to the organism comes from the diet or if it is produced by the gut microbiota, or if the vitamin derives from the transient liver storage. Hence, the model does not discriminate whether the availability of thiamine to be allocated to tissues is due to changes in the inflow of thiamine from the diet/gut microbiota or changes in the degradation rate of thiamine. Thiamine in muscles and gonads was excreted at a rate dependent on the tissue concentration (Figure 2A). Allocation of thiamine to muscles increased the probability of adult survival (Figure 2B), while allocation to gonads increased the probability of offspring recruitment (Figure 2C). Scaling of thiamine level‐dependent survival and recruitment was modeled with allometric functions that represent a fundamental feature of the biochemical reaction rate, which tends to saturate as the concentration of an enzymatic cofactor increases. The main result of the model was the temporal trend of correlations between thiamine tissue levels.

#### 2.1.2 Life History

The model was based on the life history of salmonids, in which juveniles migrate from upstream rivers to ocean feeding grounds, where they grow for a few years (~1–4 years) before migrating to spawning grounds (Jacobson et al. 2020; Karlsson and Karlström 1994). We model the life history of a female starting 1 year before first spawning (Figure 1A). Females build up gonad mass prior to spawning, and for the sake of simplicity, release eggs over 1 day. In the main part of the work, we considered iteroparous females that reproduce over several spawning seasons (cf. Figure 1A), like the Atlantic salmon that inspired the model (Crespi and Teo 2002; Persson et al. 2023). However, we also modeled females that die after spawning (see Figure S1) like semelparous salmonids such as some Pacific salmon (Crespi and Teo 2002). The model female body consists of locomotor tissues, hereafter muscles, with mass *w*_*m*_, reproductive tissues, hereafter gonads, with mass *w*_*g*_, and blood with mass *w*_*b*_ (Figure 1B). We parameterized the growth of somatic and reproductive tissues with data on temporal changes in body mass, blood volume, and gonadosomatic index in Atlantic salmon and other fish species (see Figure 1A, Appendix S1). However, the conclusions of our work hold for other growth rates and life histories with no growth after maturation, which corresponds to life histories of many mammals, birds, and invertebrates (see Figure S2). For simplicity, we assumed that tissue growth rates were independent of thiamine input level, as there is no clear relationship between female body size, in terms of somatic and gonadal mass, and thiamine status in populations of salmonids (Todisco, Fridolfsson, et al. 2024; Todisco, Hauber, et al. 2024) but see Mikkonen et al. (2011).

#### 2.1.3 Offspring Recruitment, Adult Mortality, and Reproductive Mode

Gonadal concentration of thiamine *c*_g_ at spawning determined the probability of offspring recruitment *(r)* according to

(1) $$r={\left(\frac{c_{g}}{c_{x}}\right)}^{v}$$

where *c*_x_ corresponded to the maximum concentration in tissues, set to 30 nmol/g (Todisco, Fridolfsson, et al. 2024; Todisco, Hauber, et al. 2024), and the exponent *v* determined the shape of the concentration‐dependent recruitment. Thiamine muscle level *c*_m_ set the daily survival *p* of adults according to

(2) $$p=p_{0}+\left(p_{x}-p_{0}\right){\left(\frac{c_{m}}{c_{x}}\right)}^{u}$$

with the exponent *u* that determined the shape of the concentration‐dependent survival, and *p*_0_ and *p*_x_ set the baseline daily survival at minimum and maximum thiamine muscle levels. The assumed values of *p*_0_ and *p*_x_ in the main results allowed a female with a critically low muscle level to survive 1 year with a chance of 0.001, while this chance was 0.25 for a female with muscle level *c*_x_. The conclusion of our work did not change over a wide range of baseline survival *p*_0_ and *p*_x_, consistent with the life expectancy of salmonids under natural conditions (Figure S3). In scenarios with iteroparous females, the probability of surviving the postspawning migration *p*_f_ was given by

(3) $$p_{f}={\left(\frac{c_{m}}{c_{x}}\right)}^{l}$$

And the exponent *l* determined the scaling of the concentration‐dependent survival of the postspawning migration. We assumed *l* = 0.2 in the main results, although the conclusions did not depend on the value of *l* (see Figure S4).

#### 2.1.4 Thiamine Input and Excretion

Thiamine status is a result of the balance between several processes that contribute to overall thiamine input (consumption, production by gut microbiota, digestion, assimilation, and potential loss if the vitamin is consumed as an antioxidant) and loss with a rate that depends on in‐tissue thiamine concentration (renal excretion, breakdown, see below). For simplicity, we modeled rates of input and loss, rather than reflected all component processes with their exact rates. Thiamine input, the primary characteristic of the net thiamine availability in modeled scenarios, was approximated by average thiamine concentrations in the blood *c*_b_, set in a range between 2 and 6 nmol/g. To keep our model easy to follow, we did not model the thiamine stored in the body, for example, in the liver. Instead, we assumed thiamine input levels that resulted in blood thiamine levels at the upper end of the empirical estimate range for fish. However, our conclusions did not change when we explored thiamine input scenarios resulting in blood levels close to average empirical estimates in fish, under the assumption that liver storage plays a negligible role in thiamine balance (see Figure S9). To reflect a random variation of the thiamine input, *c*_b_ varied stochastically within a range of ±2 nmol/g according to a beta distribution with a normal‐like shape (see Figure S5I). A ‘high input’ scenario of *c*_b_ = 6 nmol/g resulted in thiamine muscle levels approaching the in‐tissue concentration ceiling *c*_x_, and for a ‘low input’ scenario of *c*_b_ = 2 nmol/g, some individuals reached thiamine muscle levels considered very low even for populations that have experienced episodes of early mortality (Balk et al. 2016; Harder et al. 2020; Todisco, Fridolfsson, et al. 2024; Todisco, Hauber, et al. 2024). Here, we presented a model with thiamine input and resulting blood level *c*_b_ remaining constant throughout the season, with Appendix S1 presenting scenarios of a 2‐month starvation period prior to spawning observed in many salmonids (e.g., Vuorinen et al. 2014). Blood is often measured using the per volume unit, but here we use the per weight unit commonly used for other tissues such as muscle and gonads. However, blood has a high water content so that 1 mL can be approximated by 1 g when comparing among units.

**FIGURE 3:**
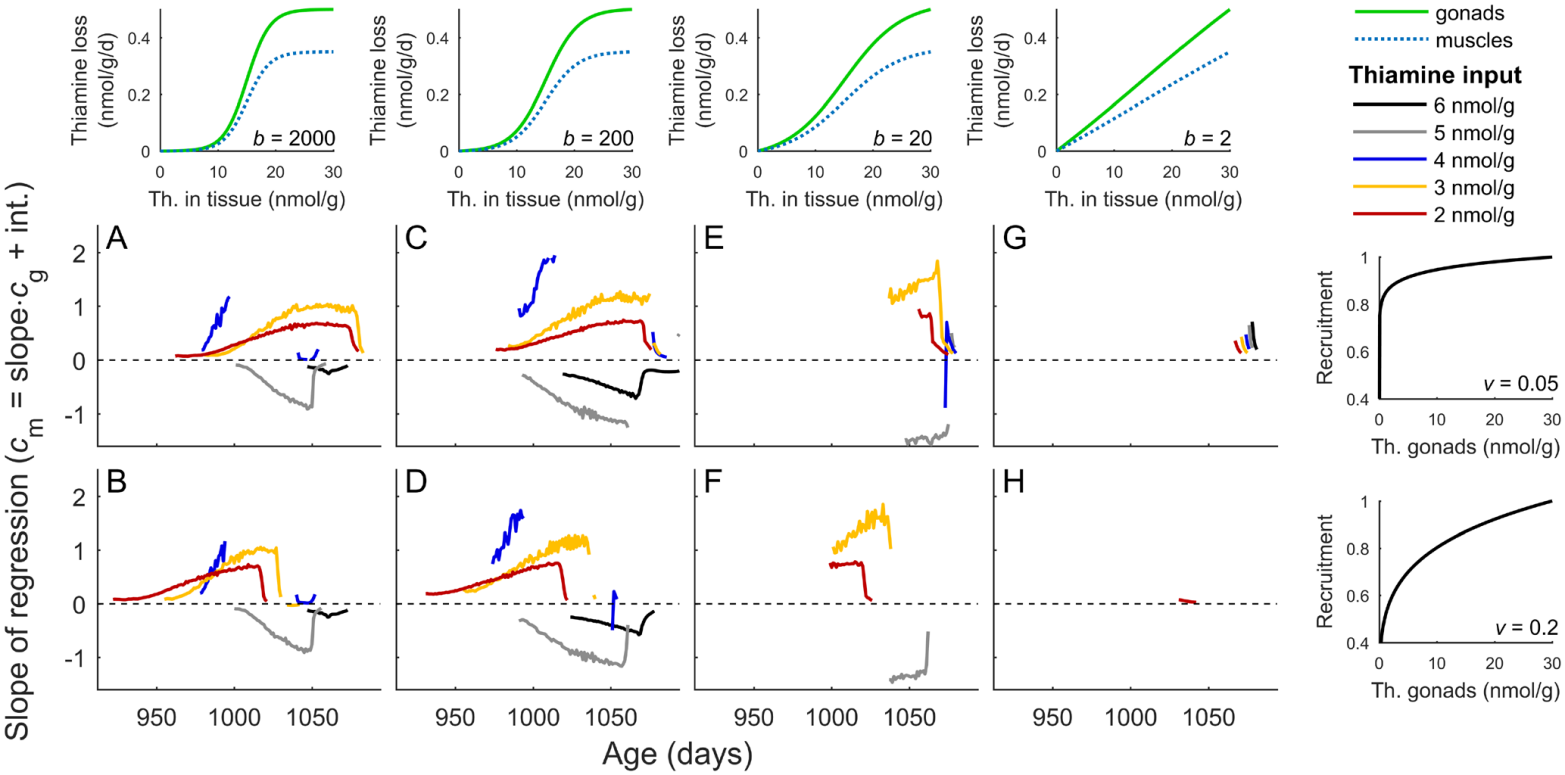
Correlation between gonad and muscle thiamine presented for different scenarios of concentration‐dependent rates of thiamine loss. (A–H) Slopes of linear regression between thiamine concentration in gonads and muscles calculated for scenarios with different thiamine input with thiamine blood level *c*_b_ (see legend), concentration‐dependent rate of thiamine loss (panels in top row), and probability of juvenile recruitment (panels in utmost right column). Thin dashed black lines demarcate positive and negative slopes of the regression calculated for thiamine content in gonads (predictor variable) and muscles (response variable). The values of the exponents *b* and *v*, which scale the concentration‐dependent rate of thiamine loss and recruitment (cf. Equations (1) and (4)), are given in the lower right corner of the panels in the top row and the utmost right column. Scenarios were modeled with scaling of the concentration‐dependent probability of survival *p* shaped by the exponent *u* = 0.05 (cf. Equation (2)). Slopes of regressions are drawn for cases with *R* ^2^ greater than 0.2.

The main mechanism of thiamine loss in our model was excretion, with active reabsorption at low plasma concentrations and active secretion at high concentrations (Gastaldi et al. 2000; Hrubša et al. 2022). With increasing concentration, the capacity of transporters for reabsorption and secretion diminishes (Shargel and Yu 2016). Therefore, at low thiamine concentrations, an increase in thiamine plasma levels is expected to result in a rate of loss that accelerates, whereas at high thiamine levels an increase in concentration is expected to result in a rate of loss that decelerates (Shargel and Yu 2016; Weber et al. 1990). For simplicity, we modeled the concentration‐dependent thiamine loss rate *k* with a sigmoidal function given by

(4) $$k=\frac{k_{x}}{b-1}\left(\frac{b+1}{b^{\left(\frac{0.5c_{x}-c}{0.5c_{x}}\right)}+1}-1\right)$$

with the shape of the function determined by the parameter *b* (see the top row panels in Figure 3). In the model the thiamine loss *k* was tissue‐specific and is given below as *k*_g_ (gonad‐specific loss) or *k*_m_ (muscle‐specific loss). The tissue concentration of thiamine *c* in Equation (4) matches thiamine in muscles *c*_m_ or gonads *c*_g_, *k*_x_ matches maximal mass‐specific rate of thiamine excretion (nmol/g/d), set to 0.5 for gonads and 0.35 for muscles, that is, the muscles TDP levels have 70% slower rate of loss than free thiamine in gonads (Figure 2A). While TDP has a slower rate of loss than free thiamine (cf. Weber et al. 1990), the conclusion from our model did not depend on changes in excretion rates *k*_x_, for either or both tissues, because an increase in excretion rate was equivalent to a decrease in thiamine input (cf. Figure S7).

Since there are no reports of thiamine overdoses in wildlife, active secretion may be of limited importance for fitness considerations. Therefore, we tested a scenario in which the thiamine excretion rate depended only on renal reuptake processes at low plasma concentrations and passive transport during filtration at intermediate and high concentrations. In this scenario, the rate of thiamine loss *k* was composed of a power and a linear function (cf. (Shargel and Yu 2016)) given by

(5) $$k=\left\{\begin{array}{c} ac^{b_{c}},\text{for}\;c<c_{t}\\ k_{x}\frac{c}{c_{x}},\text{for}\;c\geq c_{t} \end{array}\right.$$

with coefficient *b*_c_ that determined the shape of the renal reabsorption function, the *c*_t_ matched concentration threshold for passive transport during filtration only, and shape coefficients $a=\frac{c_{t}k_{x}}{c_{x}c_{t}^{b_{c}}}$ and values of *k*_x_ set to 0.5 for gonads and 0.35 for muscles (see above).

#### 2.1.5 Fitness Optimization and Forward Simulations

The model considered the evolution of allocation of thiamine to gonads and muscles by optimizing, for each day *d* in the *i*‐th year, the fraction *α*(*d*,*i*) of free thiamine pool in the blood *c*_b_ *w*_b_ that was allocated to gonads or muscles in the form of TDP (Figure 1B). The daily change of free thiamine concentration in gonads was given by

(6) $$∆c_{g}=\left(1-\alpha (d,i)\right)c_{b}w_{b}-k_{g}w_{g}$$

whereas the daily change in muscle concentration given by

(7) $$∆c_{m}=q_{m}\alpha (d,i)c_{b}w_{b}-k_{m}w_{m}$$

where *q*_m_ matches the efficiency of enzymatic conversion of free thiamine into TDP, assumed to 0.9 below, though the conclusions did not change for other *q*_m_ values (Figure S7). As only a small proportion of the thiamine found in the muscles of salmonids is in the form of thiamine monophosphate (TMP) (Koski et al. 2005), we modeled for simplicity the allocation of thiamine to the muscles as the conversion of free thiamine to TDP. Expected production of recruits for a *i*‐th year *R*_i_ was given by

(8) $$R_{i}=c_{g}\left(\alpha ,d_{s},i\right)w_{g}\left(d_{s},i\right)\prod_{d=1}^{d_{s}}p\left(c_{m},d,i\right)$$

where *d*_s_ matches the day of spawning. The fitness in our model was approximated by the expected lifetime production of recruits *V* given by

(9) $$V=R_{i=1}\left(\alpha \right)+\sum_{i=2}^{\infty }p_{f}\left(c_{m},d_{s},i\right)R_{i}\left(\alpha \right)$$

where *p*_f_ matches probability of surviving the postspawning migration. We used backward optimization (cf. Clark and Mangel 2000) to find the optimal strategy of thiamine allocation to gonads and muscles *α* with maximum fitness given by Equation (9) and assuming *i*_max_ = 15. Next, we simulated populations of *N* = 1000 females that followed the state‐dependent optimal strategy *α* obtained with backward optimization, with initial thiamine levels of *c*_g_ = 0 and *c*_m_ = 15 nmol/g. Population data were used to calculate correlations between thiamine tissue levels. To reduce the effect of round‐off numerical errors, correlations were calculated for females with gonadal thiamine *c*_g_ > 0, only for populations with variation greater than 0.5 nmol, and more than 2/3 of the females had *c*_g_ > 0. All calculations were done in MATLAB.

### 2.2 Empirical Data

We contrasted the model predictions with the outcomes of the empirical analysis, in which we measured and correlated thiamine concentrations in the muscles and gonads of salmon sampled in the North Atlantic and the Baltic Sea (see below for details). Specifically, we sampled Atlantic salmon that spend their marine feeding period in the Baltic Sea and in the North Atlantic Ocean to study the allocation of thiamine to locomotor (muscles) and reproductive tissues (gonads) in two different systems. While there are no records of thiamine deficiency among North Atlantic populations inhabiting Norwegian and Swedish west coast rivers, salmon populations in the Baltic Sea regularly display periods of deficiency (Bengtsson et al. 1999; Majaneva et al. 2020; Todisco, Fridolfsson, et al. 2024; Todisco, Hauber, et al. 2024; Vuorinen et al. 2021). The same fish have been studied in terms of thiamine dynamics during different life phases (Todisco, Fridolfsson, et al. 2024; Todisco, Hauber, et al. 2024), and that study details the methods of catching, sampling, and thiamine estimation. In short, salmon were caught and sampled according to applicable international, national, and/or institutional guidelines (for ethical permissions, see Todisco, Fridolfsson, et al. 2024; Todisco, Hauber, et al. 2024). Sampling took place in the Southern Baltic Sea (main feeding area), in river mouths, and then upstream after the river migration and later on in conjunction with spawning in the same rivers (Baltic populations: Torne, Luleå, and Umeå rivers; North Atlantic populations: Drammen, Driva, Ätran, and Enningdal rivers). Thiamine was quantified according to Brown et al. (1998) with modifications according to Vuorinen et al. (2002), Futia et al. (2017), and Futia and Rinchard (2019). Thiamine concentrations were similar among rivers within the two systems (i.e., within Baltic versus North Atlantic populations; Todisco, Fridolfsson, et al. 2024; Todisco, Hauber, et al. 2024). Hence, the estimates of thiamine in salmon expressed as amount per wet weight were pooled within systems, and correlations between muscle and gonad tissues were performed using Spearman rank tests.

## 3 Results

The allocation strategy of the vitamin to muscles and gonads depended on the assumed thiamine input (Figure 1B), the concentration‐dependent rate of thiamine loss (Figure 2A), and probabilities of adult survival and juvenile recruitment (Figure 2B,C). At low thiamine input, muscles and gonads have lower thiamine concentrations than in the scenario with a high input (Figure 2D,E). Prior to spawning, model females allocated available thiamine to muscles and gradually switched allocation to gonads, with a complete switch of allocation of all available thiamine to gonads occurring a couple of weeks before spawning (Figure 2D,E). Due to the stochastic variation in daily input, females differed in thiamine tissue levels (Figure 2D,E), which was used to analyze correlations between levels in gonads and muscles (Figure 2F–R). The gradual increase in the amount of allocated thiamine to the gonads resulted in a positive correlation between thiamine levels in gonads and muscles for low thiamine input (Figure 2F–K), and a negative correlation for the high thiamine input scenario (Figure 2L–R). The same pattern was observed for correlations between gonadal and muscle thiamine in scenarios with a two‐month period of starvation prior to spawning (Figures S5 and S6), although in these scenarios gonadal thiamine concentrations decreased over the starvation period (Figure 2D,E versus Figure S4A,B).

The correlations between thiamine gonadal and muscle levels were observed only when the rate of loss, with involved mechanisms of reabsorption and secretion (cf. Equation (4)), changed in a nonlinear fashion with concentration (Figure 3). A linear increase in the rate of loss with concentration (Figure 3G,H) resulted in no correlation due to a rapid shift in allocation from muscles to gonads. A positive correlation in thiamine concentration between gonads and muscles, with a regression slope that increases towards spawning, was observed at low thiamine input where thiamine muscle levels were relatively low (Figure 2D,F–K). In these scenarios, thiamine input was a limiting factor of survival and recruitment, allowed for sustaining only low muscle levels (Figure 2D), and an individual with higher than average thiamine status of the muscles would minimize overall thiamine loss by allocating more of the available thiamine to the gonads (see blue circles in Figure 2A,D). In high thiamine input scenarios, muscle levels were very high (Figure 2E), and the higher the thiamine muscle level of an individual, the less the rate of thiamine loss increases with allocation to muscles (see blue diamonds in Figure 2A,E). Individuals with high thiamine muscle levels could increase their survival at relatively low cost in terms of thiamine loss or allocate to the gonads, reducing thiamine loss but at the expense of survival probability. Thus, for a high thiamine input, concentration in gonads correlated negatively with muscle thiamine content (Figure 2D,F–K). In the case of allocation to the gonads early in the season and a high thiamine input, small gonadal mass would result in a rapid build‐up of gonadal thiamine level and a rapid increase in the rate of thiamine loss from gonads (Figure 2A,E). Therefore, in scenarios with high thiamine input, allocation to the gonads started when the gonads were larger, that is, later in the season in comparison to low input scenarios (Figure 2D vs. Figure 2E).

**FIGURE 4:**
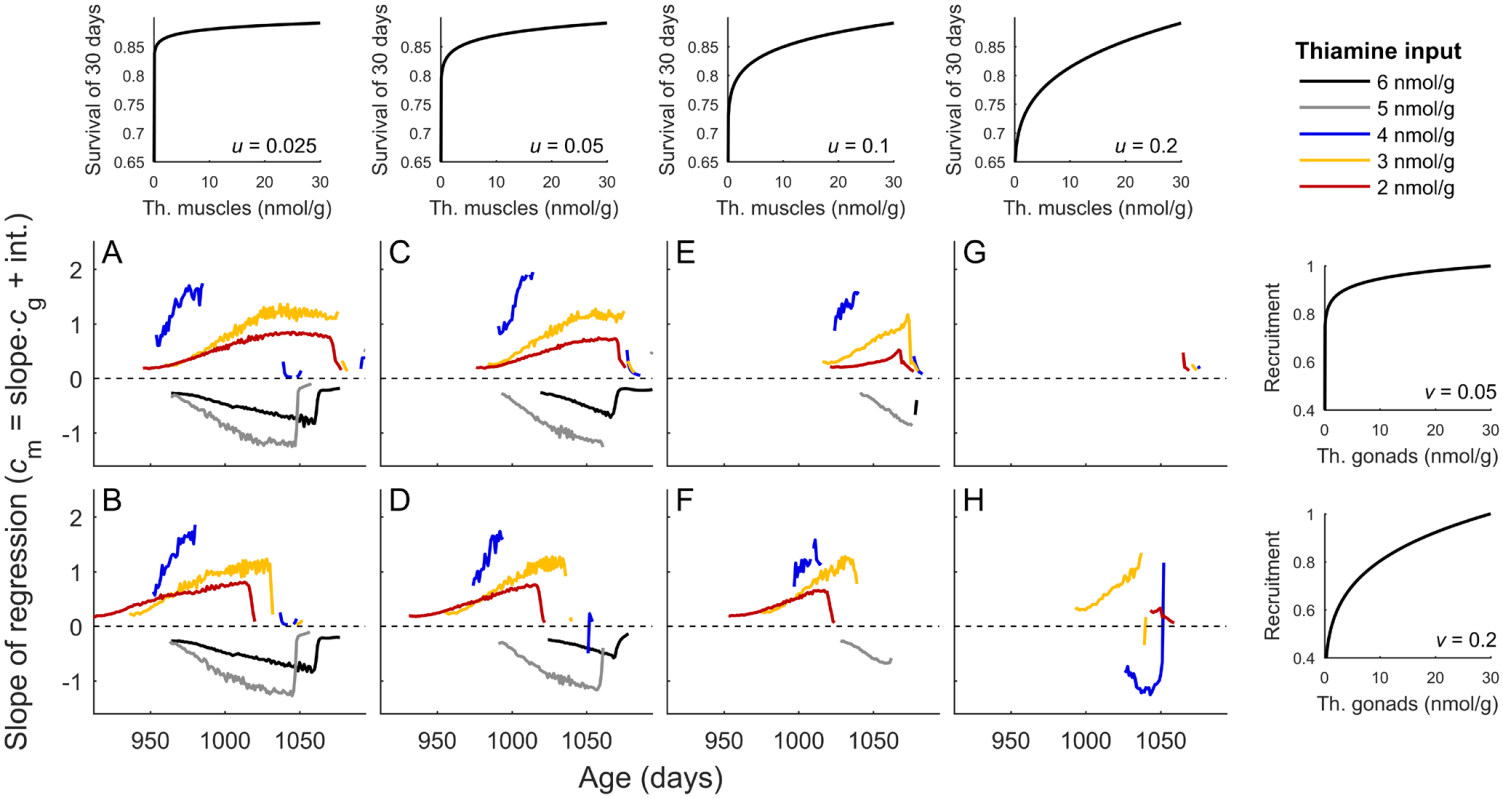
Correlation between gonad and muscle thiamine presented for different scenarios of concentration‐dependent rates of adult survival and juvenile recruitment. (A–H) Slopes of linear regression between thiamine concentration in gonads and muscles calculated for scenarios with different thiamine input with thiamine blood level *c*_b_ (see legend), scaling of thiamine‐dependent adult survival (panels in the top row), and probability of juvenile recruitment (panels in utmost right column). Thin dashed black line demarcates positive and negative slopes of the regression calculated for thiamine content in gonads (predictor variable) and muscles (response variable). The values of the exponents *u* and *v*, which scale the concentration‐dependent adult survival and recruitment (cf. Equations (1) and (2)), are given in the lower right corner of the panels in the top row and the utmost right column. Scenarios were modeled with scaling of the concentration‐dependent rate of thiamine loss shaped by the exponent *b* = 200 (cf. Equation (4)). Slopes of regressions are drawn for cases with *R* ^2^ greater than 0.2.

The selection to allocate thiamine in a way that reduced losses due to excretion depended on the concentration‐dependent scaling of fitness components, that is, adult survival and juvenile recruitment (Figure 4, Figure S8). A strong relationship between the degree to which adult survival changed along with the concentration in muscles (Figure 4, column‐wise) led to a rapid switch of allocation from muscles to gonads shortly before spawning, and no correlations (Figure 4G,H). The stronger the increase in juvenile recruitment with thiamine gonad level, the more pronounced the correlations caused by the gradual shift in thiamine allocation from muscles to gonads (Figure 4, Figure S8 row‐wise). Similarly, in the model of semelparous reproduction, a positive correlation between thiamine in gonads and muscles occurred in low input scenarios when juvenile recruitment, but not adult survival, increased strongly with thiamine tissue levels (Figure S1). At high and intermediate thiamine inputs, semelparous females abruptly switched allocation from muscles to gonads, which led to no correlations (Figure S1).

**FIGURE 5:**
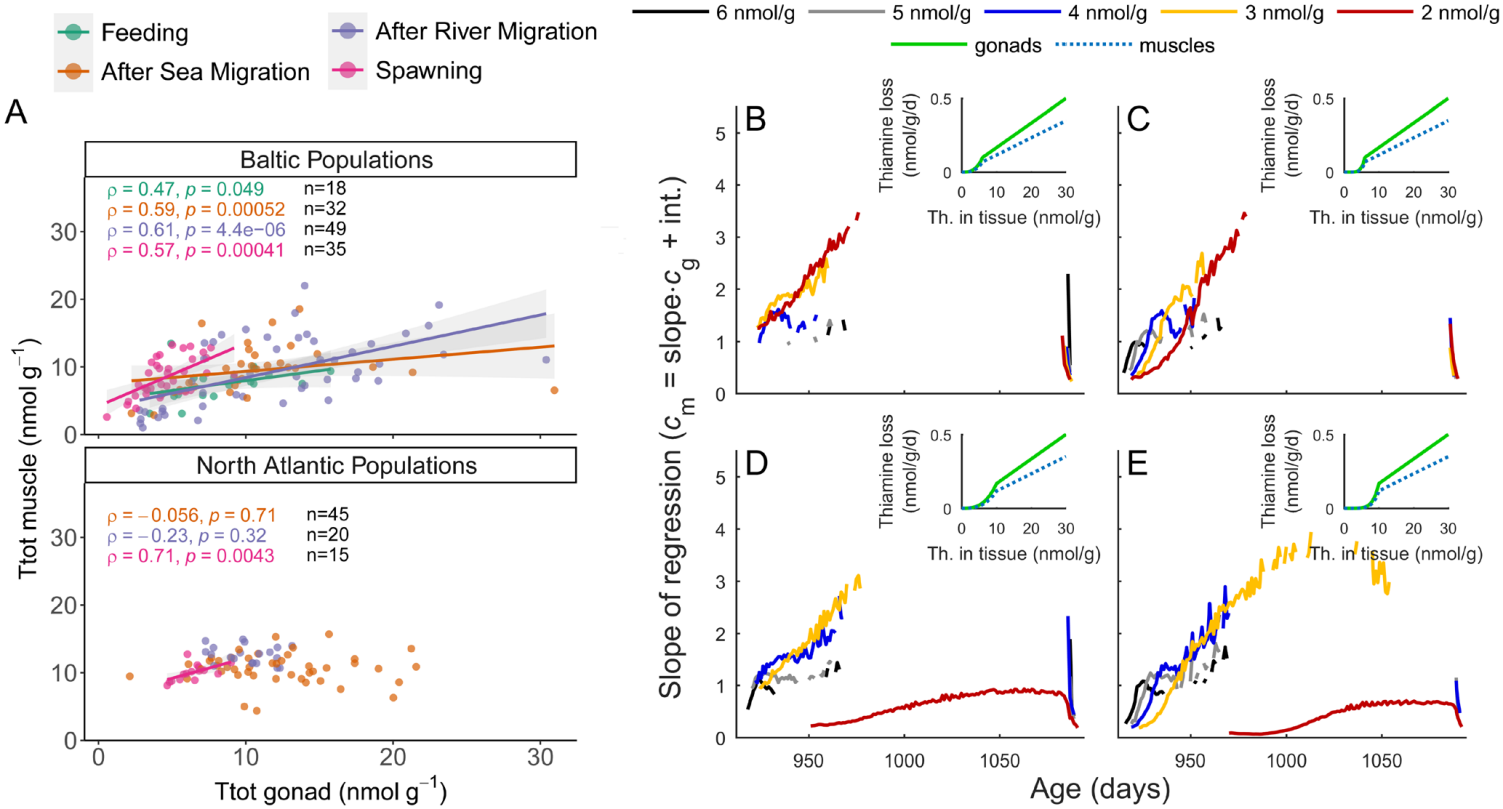
Correlation between thiamine levels in gonads and muscles derived from empirical data and the theoretical model. (A) Correlation between the concentration of total thiamine (TF + TMP + TDP) in gonads and muscles of female Atlantic salmon. Females were sampled from the Baltic Sea population, which has been characterized by numerous episodes of thiamine deficiency in recent decades, and the North Atlantic population, which has no known cases of thiamine deficiency. Females were sampled at the subsequent life stages prior to spawning (see legend). (B–E) Predictions of the model with the concentration‐dependent rate of thiamine loss composed of a power function approximating renal uptake of thiamine at low concentrations and a linear function approximating excretion by passive transport only at higher concentrations. The shape parameters of the example scenarios of concentration‐dependent thiamine loss *k*_c_ (cf. Equation (5)) are as follows (B–D) *b*_c_ = 3, (C–E) *b*_c_ = 6; (B, C) *c*_t_ = 6, (D, E) *c*_t_ = 10. Scenarios were modeled with the concentration‐dependent probability of survival *p* shaped by the exponent *u* = 0.025 (cf. Equation (2)), and the probability of juvenile recruitment shaped by the *v* = 0.025 (cf. Equation (1)). Slopes of regressions are drawn for cases with *R* ^2^ greater than 0.2.

Analysis of empirical data for Baltic salmon, a population that has experienced numerous episodes of early mortality and thiamine deficiency, showed a positive correlation between thiamine levels in gonads and muscles, whereas females sampled in the North Atlantic did not show this pattern (Figure 5A). The positive correlation with a slope that tended to increase over life stages in Baltic salmon, and the lack of support for correlations in the North Atlantic population, was consistent with predictions of the model in which thiamine loss was determined solely by the mechanisms of active reabsorption and passive transport (Figure 5B,C, Equation (5)).

## 4 Discussion

Here we modeled the evolution of allocation strategies of a key metabolic micronutrient to investigate mechanisms underlying correlations between the vitamin levels in gonads and muscles. The model, based on a combination of life history trade‐offs and physiological mechanisms of micronutrient loss, revealed that a positive correlation between thiamine levels in gonads and muscles is an indicator that females are constrained by thiamine input. This prediction was consistent with empirical data analysis of thiamine tissue levels in Atlantic salmon populations. The positive correlations between gonadal and muscle levels, with an increasing slope of the regression over the spawning season, were found in Baltic Sea females but not in North Atlantic females (Figure 5), which is consistent with the history of thiamine deficiency detected only in the Baltic Sea populations (Bengtsson et al. 1999; Keinänen et al. 2012; Todisco, Fridolfsson, et al. 2024; Todisco, Hauber, et al. 2024; Vuorinen et al. 2021). The model showed that strategies of micronutrient allocation evolve to optimize fitness effects of gonadal and muscle levels and to minimize losses due to excretion. The thiamine concentrations computed by the model are in the range of previously published empirical estimates (Balk et al. 2016; Keinanen et al. 2022; Todisco, Fridolfsson, et al. 2024; Todisco, Hauber, et al. 2024; Vuorinen et al. 2021) but should not be directly compared but rather used to understand evolutionary and ecological mechanisms regulating thiamine dynamics in aquatic top predators, such as salmon.

The positive correlations between gonadal and muscle levels at low thiamine input were found only when reabsorption affected the rate of thiamine loss (Figure 3 and Figure 5B–E). Active reabsorption of organic ions is essential for maintaining physiological homeostasis in all vertebrates, including fish (Beyenbach 2004). The mechanisms of thiamine reabsorption are well understood in mammals (Gastaldi et al. 2000; Hrubša et al. 2022; Weber et al. 1990), but have not been studied in fish to our knowledge. The positive correlations were found at low thiamine input, regardless of whether active secretion contributed to the overall rate of thiamine loss (see e.g., Figure 3A,D vs. Figure 5D–E). For low thiamine input, muscle levels were low to intermediate, and a further allocation to muscles not only increased survival but also the rate of loss in an accelerating fashion (Figure 2A,D). Allocation to the gonads in this situation reduced the rate of loss as long as gonadal concentration was low (Figure 2A,D). For high input scenarios, muscle levels were higher, and rates of loss at such high tissue levels similarly depended on whether excretion was dominated by active secretion or passive transport only (Equation (4) vs. Equation (5)). For the model with active secretion, that is, a slowing increase in the rate of loss at high concentration, allocation to gonads could reduce the rate of loss but also reduce survival (cf. Equation (2)). Allocation to the muscles slowed the increase in the rate of excretion and provided fitness benefits in terms of a higher adult survival (Figure 2A,B,E). Thus, females with higher than average thiamine status allocated thiamine to muscles to a greater extent than females with lower than average thiamine status (Figure 2A,E), resulting in a negative correlation between gonadal and muscle levels. However, the negative correlations were absent if loss rates were determined by passive transport only, that is, a linear increase in loss rate with concentration. In scenarios with passive transport only, a reduction in thiamine loss due to allocation to the gonads did not outweigh a reduction in survival caused by lowered muscle concentration. As a result, females rapidly shifted thiamine allocation to the gonads shortly before spawning, and tissue levels did not correlate (e.g., Figure 3G,H, Figure 5B–E). Analyses of empirical data did not show negative correlations between thiamine levels in gonads and muscles (Figure 5A,B). Pharmacokinetic studies in salmon are rare and lack quantification of the passive transport or active secretion rates (Koski et al. 2005). Active secretion plays an important role in the removal of organic ions by fish (Beyenbach 2004), and the lack of correlation in empirical data may be due to thiamine levels being too low under natural conditions for active secretion to be the main determinant of excretion rates.

In our model, a strategy to minimize thiamine loss by early filling the gonads with thiamine was optimal only when adult survival did not decline sharply with decreasing muscle levels (e.g., Figure 4G,H, Figure S3). A reduction in muscle levels had a propagating negative effect on life expectancy, as daily survival probabilities multiply. This was likely due to strong selection to maintain muscle levels relatively high, which could only be offset if early allocation to the gonads significantly reduced thiamine loss (Figures 3 and 4). Consistent with this, in semelparous life histories, where muscle levels determine only current reproduction in a spawning season, the tendency to minimize thiamine losses by an early and gradual shift of allocation to the gonads was relatively weak and only detected in scenarios with low thiamine input and strong scaling of recruitment with gonadal level.

Despite the impact of thiamine deficiency on wildlife (Gilbert 2018; Sutherland et al. 2018), our understanding of the ultimate drivers and ability to offset the negative aspects of mass juvenile mortality is limited. Studies of flagship top consumers affected by early mortality due to thiamine deficiency show a highly variable thiamine status of females within populations and along the life cycle (e.g., Todisco, Fridolfsson, et al. 2024; Todisco, Hauber, et al. 2024; Vuorinen et al. 2020). The detection of a positive correlation between levels in locomotor and reproductive tissues may be a complementary sign of limited thiamine availability, reducing the effects of stochastic variability as each point in the analyses only represents a single individual. In our work, we made several assumptions for the sake of simplicity or to keep the model results general. For example, many salmonids, including Atlantic salmon, stop feeding a few weeks or many months before spawning (Foldvik et al. 2024; Vuorinen et al. 2014). In these species, thiamine levels in the gonads decrease while the gonads enlarge prior to spawning (Todisco, Fridolfsson, et al. 2024; Todisco, Hauber, et al. 2024). In our work, we tested scenarios with and without prespawn starvation, although a decrease in thiamine levels over time was only detected in scenarios where females did not forage prior to spawning (Figure S4A,B vs. Figure 2D,E). However, the main conclusion of our work did not change for the assumed prespawn starvation. Similarly, we modeled iteroparous life histories, although life histories of semelparous salmonids were also considered (Figure S1). The positive correlations between thiamine concentration in gonads and muscles were indicative of limited thiamine input in both life history types adopted by salmonids (Crespi and Teo 2002; Persson et al. 2023). The positive correlation between gonad and muscle levels was also indicative of low thiamine input in scenarios where female muscle mass did not change (Figure S2), which corresponds to the life histories of capital breeders that do not grow after maturation, such as many mammals, birds, and invertebrates (e.g., Ejsmond and Ejsmond 2022; Houston et al. 2006; Varpe et al. 2009). However, we modeled salmonids that are capital breeders, that is, build up reserves for reproduction in the future. It is unclear if the conclusion of our work holds for income breeders, that is, taxa that fuel reproduction with concurrent feeding. This is because capital and income breeders are often subject to a different nature of trade‐off between current and future reproduction (e.g., Ejsmond et al. 2018; Houston et al. 2006; Sainmont et al. 2014), which was a key driver of the results in our work.

The role of spatiotemporal variation in macronutrients availability is well recognized in life history evolution and constitutes a major advance in our understanding of the system (e.g., Filipiak and Weiner 2014; Snell‐Rood et al. 2015; Swanson et al. 2016). The question of how life histories are shaped by micronutrient availability is rarely asked, with the existing theory dealing with ‘resources’ being allocated between competing needs (e.g., Ejsmond et al. 2023; Johansson et al. 2018; Kozlowski 2006; Stearns 1992). Our work shows that the framework of optimal allocation to competing needs and fitness maximization can be used to translate classic life history trade‐offs (cf. Williams 1966) to dilemmas of optimal allocation of limited micronutrients. The expectation of selection pressure due to the limitations by availability of essential micronutrients stems from the reported deficiency‐related dysfunctions in neural signaling, growth, and survival of top consumers worldwide. However, there are important features of the micronutrient allocation that may cause the conclusions of this study to depart from an existing theory of life history evolution based on optimal resource allocation. In resource allocation models, the rate of food acquisition is often assumed to change with an allocation strategy, which is often key to the predictions of these models (Ejsmond et al. 2015; Heino and Kaitala 1999; Jørgensen et al. 2006; Taborsky et al. 2003; Thygesen et al. 2005). For the sake of simplicity, we assumed that the micronutrient input does not change with the allocation strategy and does not change with the muscle concentrations. We believe that this assumption was made not only for simplicity but may also reflect the fundamental property of the thiamine deficiencies in ecosystems, where low micronutrient availability is often believed to be a general feature of the environment (cf. Ejsmond et al. 2019; Fitzsimons et al. 1999; Keinänen et al. 2012). Low availability could be due to inefficient food webs with thiamine loss in each trophic stage, low absolute amount of thiamine in food resource, low amount of thiamine in relation to other nutrients, low uptake due to degradation of thiamine in the GI track (e.g., due to thiaminase), or high loss of thiamine due to degradation of the vitamin during lipid metabolism (see introduction). The presented framework provides a platform that could be extended to study the micronutrient allocation strategies in environments with a spatial variation of macronutrient availability and behavioral choices that improve thiamine intake by diet selection, the role of TDP as antioxidant in preventing oxidative stress, and/or other factors. These research avenues are novel and may provide a long‐awaited breakthrough in seeking the drivers of micronutrient limitation and understanding the causes of episodic thiamine deficiencies in coastal and marine ecosystems worldwide.

## Author Contributions

**Maciej Jan Ejsmond:** conceptualization (equal), data curation (lead), formal analysis (lead), funding acquisition (supporting), investigation (equal), supervision (equal), validation (lead), visualization (lead), writing – original draft (lead). **Vittoria Todisco:** conceptualization (equal), data curation (equal), formal analysis (equal), investigation (lead), validation (equal), visualization (equal), writing – original draft (supporting), writing – review and editing (equal). **Marc M. Hauber:** conceptualization (supporting), investigation (supporting), validation (equal), writing – review and editing (equal). **Kjetil Hindar:** conceptualization (supporting), funding acquisition (supporting), investigation (equal), supervision (supporting), validation (supporting), writing – review and editing (equal). **Samuel Hylander:** conceptualization (equal), funding acquisition (lead), project administration (lead), resources (lead), supervision (lead), validation (equal), writing – original draft (supporting), writing – review and editing (equal).

## Conflicts of Interest

The authors declare no conflicts of interest.

## Supporting information


**Appendix S1:** Sensitivity analysis of the model results.

## Data Availability

The code and raw data are available at the Zenodo open digital repository https://doi.org/10.5281/zenodo.13826262.
