# Supplementary material for: Physiological Mechanisms and Life History Trade‐Offs in Salmonids Shape In‐Tissue Correlations of an Essential Micronutrient
Source: Ecol Evol. 2025 Oct 16;15(10):e72339. doi: 10.1002/ece3.72339 (PMC12530007; doi:10.1002/ece3.72339)
Supplement: Supplementary file 1 — Appendix S1: Sensitivity analysis of the model results. [file ECE3-15-e72339-s001.docx]

**Appendix S1. Sensitivity analysis of the model results**

**Table of contents**

**1. *Micronutrient allocation in semelparous organisms* 2**

**2. *Micronutrient allocation in determinate growers* 3**

**3. *Sensitivity of results to baseline survival rates* 5**

**4. *Survival of post-spawning migration dependent on thiamine levels* 6**

**5. *Starvation prior to spawning* 8**

**6. *Tissue-specific thiamine loss rates and conversion efficiency of free thiamine to TDP* 10**

**7. *Extended version of Fig 4 from the main manuscript* 11**

**8. *Parameterisation of temporal changes in tissue mass*  12**

**9. *Simulations with low thiamine input and low excretion rates* 12**

**10. *References* 14**

**1. *Micronutrient allocation in semelparous organisms*** In the main manuscript we present results for iteroparous life histories with females able to repeat spawning. In Fig S1 we present results for semelparous life histories, as in many salmonid species females die after spawning (see Discussion in the main manuscript).
**
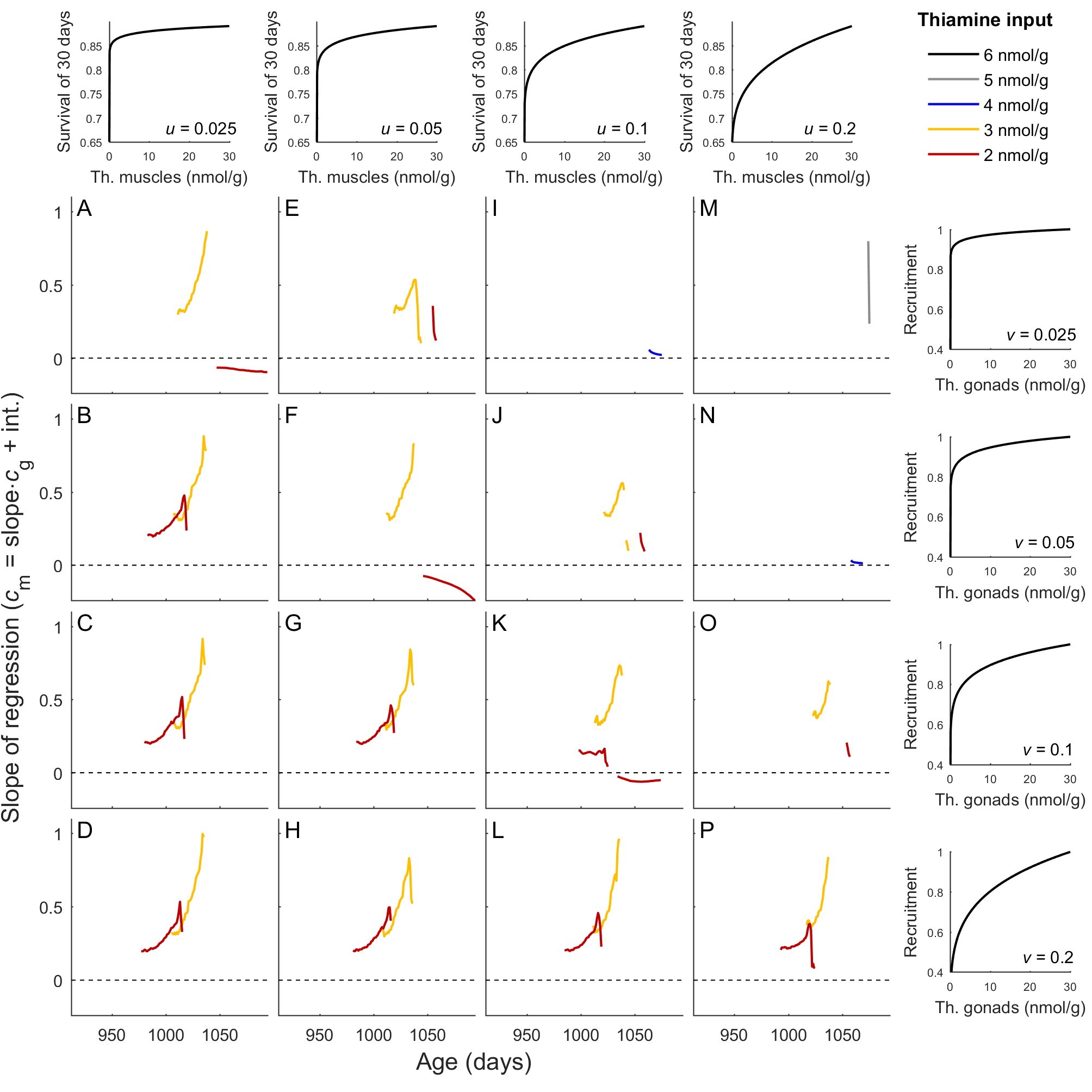
**
Fig S1. Correlation between gonad and muscle thiamine level in semelparous females (dying after spawning), presented for different scenarios of concentration-dependent rates of adult survival and juvenile recruitment. The strategy of optimal allocation of micronutrients to gonads and muscles has been optimized for females in which spawning is terminated by death. (A-P) Slopes of linear regression between thiamine concentration in gonads and muscles calculated for scenarios with different thiamine input levels *c*_b_ (see legend), scaling of thiamine-dependent adult survival (panels in the top row), and probability of juvenile recruitment (panels in utmost right column). Thin dashed black lines demarcates positive and negative slopes of the regression calculated for thiamine content in gonads (predictor variable) and muscles (response variable). The values of the exponents *u* and *v*, which scale the concentration-dependent adult survival and recruitment (see eq. 1 and eq. 2 in the main text), are given in the lower right corner of the panels in the top row and the utmost right column. Scenarios were modelled with scaling of the concentration-dependent rate of thiamine loss shaped by the exponent *b*=200 (see eq. 4 in the main text). Regressions slopes are plotted for cases with R^2^ greater than 0.2 and gonad thiamine variation greater than 0.5 nmol.

**2. *Micronutrient allocation in determinate growers***

In the main manuscript we present results for indeterminate growers with females that continue to grow after maturation. In Fig S2 we present results for determinate growing females in which somatic tissue mass does not change after maturation.


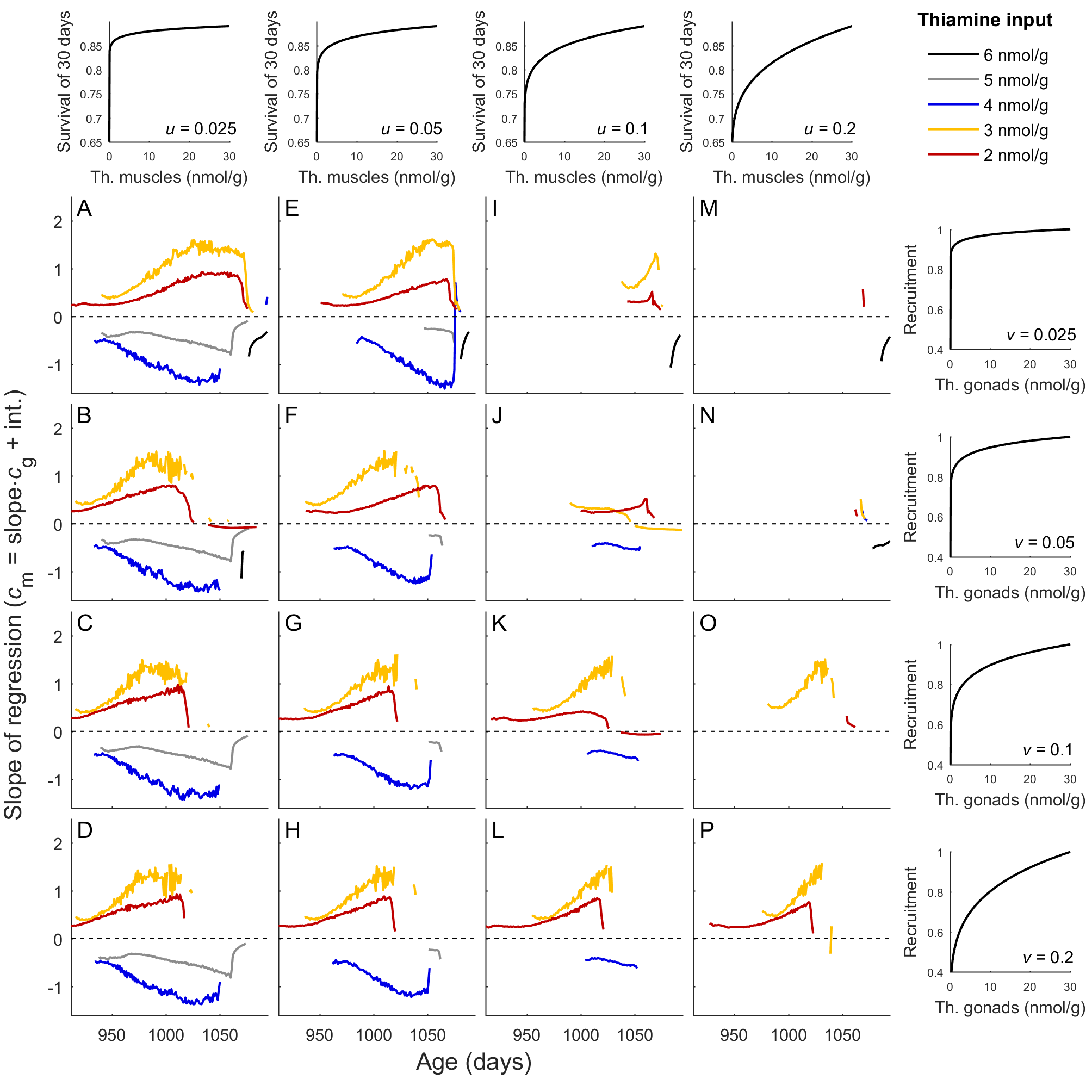

Fig S2. Correlation between gonad and muscle thiamine in determinate growing females, i.e. with constant somatic tissue mass, presented for different scenarios of concentration-dependent adult survival and juvenile recruitment. The mass of somatic tissues of females was set to 5 kg. (A-P) Slopes of linear regression between thiamine concentration in gonads and muscles calculated for scenarios with different thiamine input levels *c*_b_ (see legend), scaling of thiamine-dependent adult survival (panels in the top row), and probability of juvenile recruitment (panels in utmost right column). Thin dashed black lines demarcates positive and negative slopes of the regression calculated for thiamine content in gonads (predictor variable) and muscles (response variable). The values of the exponents *u* and *v*, which scale the concentration-dependent adult survival and recruitment (see eq. 1 and eq. 2 in the main text), are given in the lower right corner of the panels in the top row and the utmost right column. Scenarios were modelled with scaling of the concentration-dependent rate of thiamine loss shaped by the exponent *b*=200 (see eq. 4 in the main text). Regressions slopes are plotted for cases with R^2^ greater than 0.2 and gonad thiamine variation greater than 0.5 nmol.

**3. *Sensitivity of results to baseline survival rates***


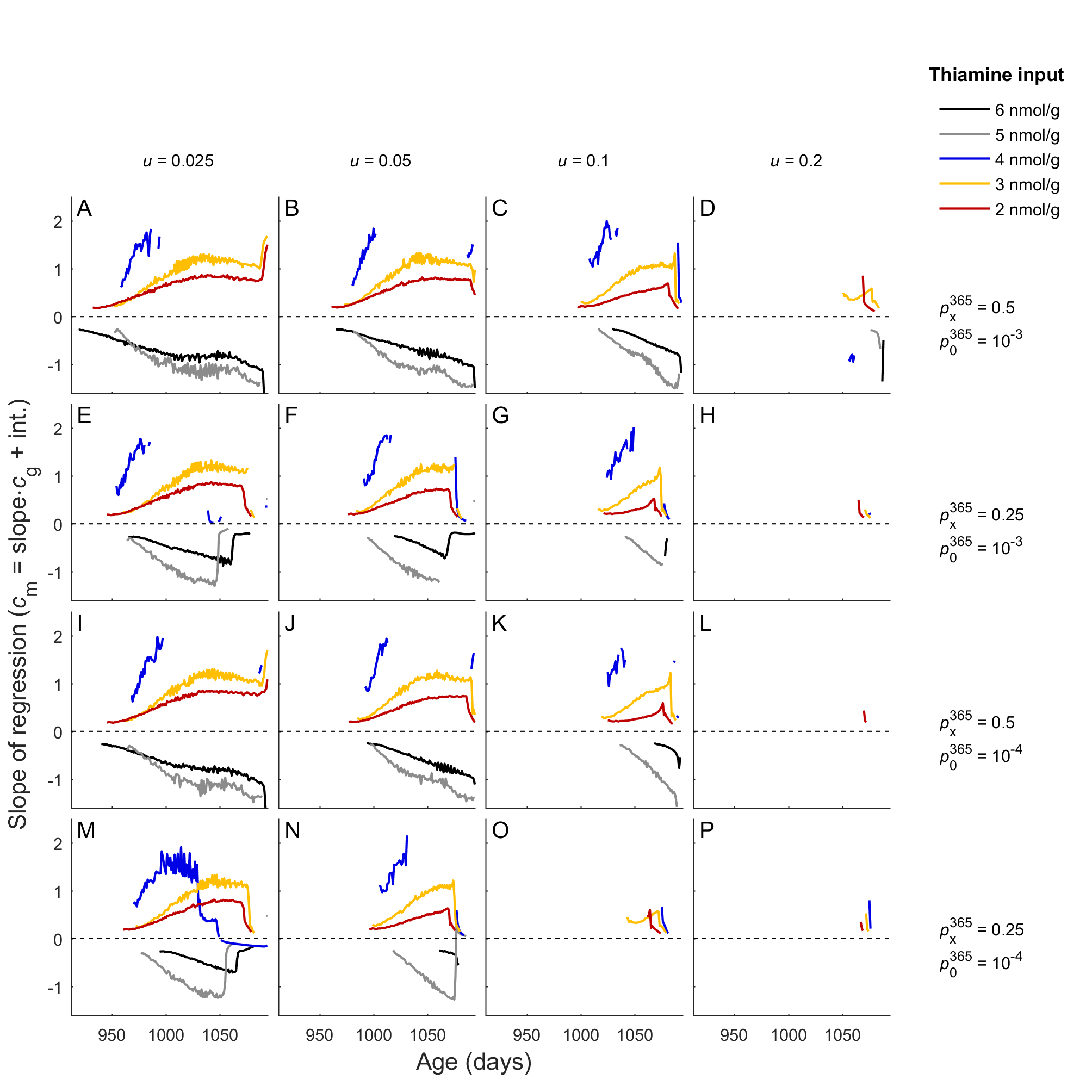

Fig S3. Correlation between gonad and muscle thiamine for four combinations of the baseline survival rates. (A-P) Slopes of linear regression between thiamine concentration in gonads and muscles calculated for scenarios with different thiamine input levels *c*_b_ (see legend), scaling of thiamine-dependent adult survival (the exponent *u* that scales the concentration-dependent adult survival given in the top row), and the baseline daily survival probabilities *p*_0_ and *p*_x_ (see the utmost right column). The *p*_0_ and *p*_x_ set the daily survival at minimum and maximum thiamine muscle levels (see eq. 2 in the main text). Scenarios presented in the main results are shown in E-H. Thin dashed black lines demarcates positive and negative slopes of the regression calculated for thiamine content in gonads (predictor variable) and muscles (response variable). Scenarios were modelled with the concentration-dependent rate of thiamine loss shaped by the exponent *b*=200 (see eq. 4 in the main text), and the concentration-dependent juvenile recruitment *r* shaped by *v*=0.05 (see eq. 1 in the main text). Regressions slopes are plotted for cases with R^2^ greater than 0.2 and gonad thiamine variation greater than 0.5 nmol.

**4. *Survival of post-spawning migration dependent on thiamine levels***

In our model, the probability *p*_f_ that an iteroparous female survives the post-spawning migration depends on the muscle thiamine level (see eq. 3 in the main text). A value of the exponent *l* close to 0 corresponds to a weak dependence of *p*_f_ on muscle thiamine levels, whereas *l* close to 1 sets a strong dependence. In the main manuscript we present results for a weak dependence between thiamine muscle level and the probability of the post-spawning migration with *l*=0.2 (Fig 4, Fig S8). In Fig S4 we present results for *l*=0.8 i.e. a strong dependence between thiamine level and the survival probability of post-spawning migration *p*_f_.


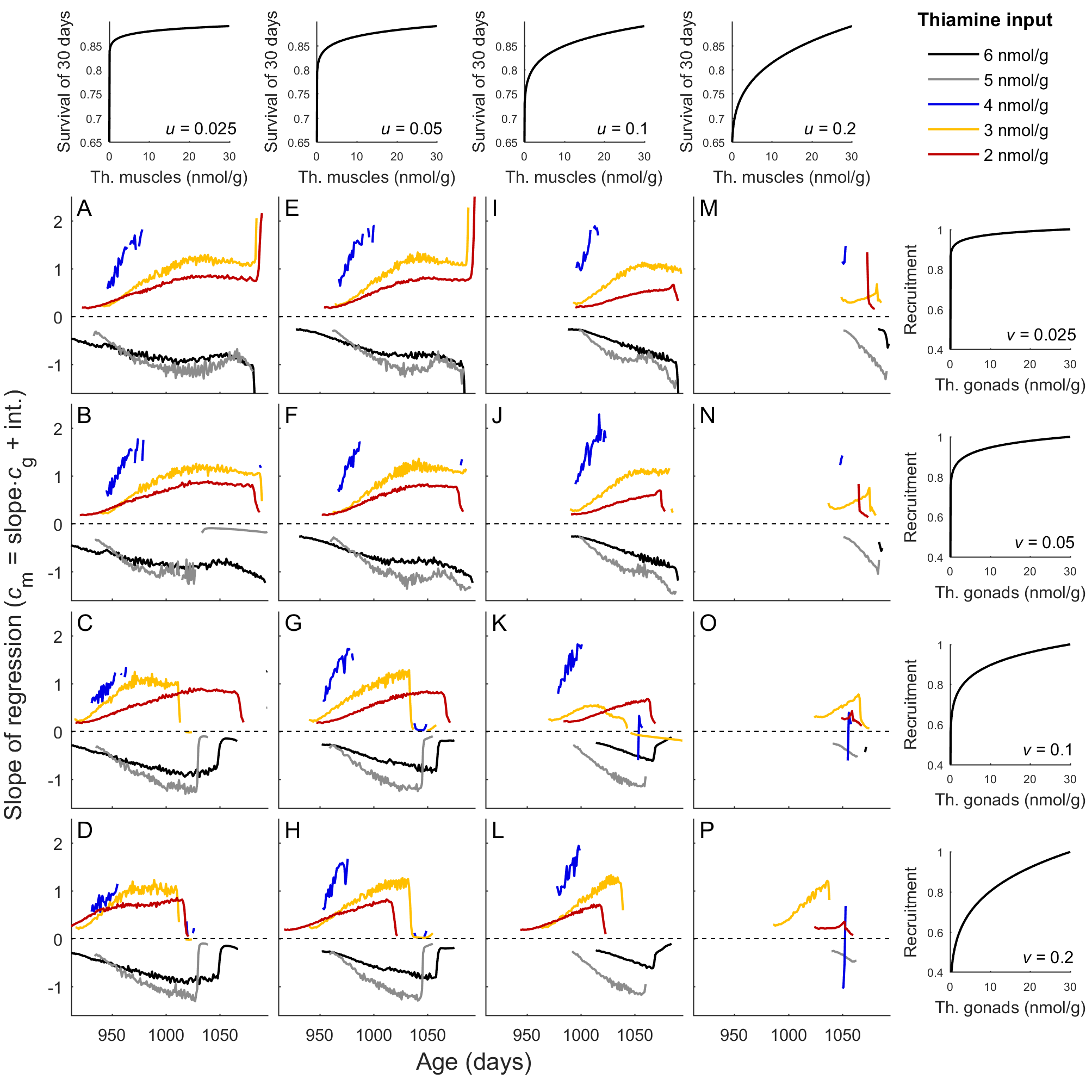

Fig S4. Correlation between gonad and muscle thiamine for a strong dependence between thiamine concentration and the survival probability of post-spawning migration. Correlations are presented for different scenarios of concentration-dependent rates of adult survival and juvenile recruitment, and post-spawning migration survival *p*_f_ set by the exponent *l*=0.8. (A-P) Slopes of linear regression between thiamine concentration in gonads and muscles calculated for scenarios with different thiamine input levels *c*_b_ (see legend), scaling of thiamine-dependent adult survival (panels in the top row), and probability of juvenile recruitment (panels in utmost right column). Thin dashed black lines demarcates positive and negative slopes of the regression calculated for thiamine content in gonads (predictor variable) and muscles (response variable). The values of the exponents *u* and *v*, which scale the concentration-dependent adult survival and recruitment (see eq. 1 and eq. 2 in the main text), are given in the lower right corner of the panels in the top row and the utmost right column. Scenarios were modelled with scaling of the concentration-dependent rate of thiamine loss shaped by the exponent *b*=200 (see eq. 4 in the main text). Regressions slopes are plotted for cases with R^2^ greater than 0.2 and gonad thiamine variation greater than 0.5 nmol.

**5. *Starvation prior to spawning***

In the pre-spawn starvation scenarios, dietary thiamine intake ceases two months before spawning and we assume that thiamine input decrease exponentially due to microbiota production, vitamin degradation processes, and mobilization of thiamine stored in the liver (Koski et al. 2005). The thiamine input level *c* starts to decrease on day *d*_s_=0, i.e. 60 days before spawning, and reaches the lowest level one day before spawning according to
(S.1) $c(d_{s})={c_{b}e}^{-zd_{s}}$
with the rate of decrease over time determined by the exponent *z*. The results of the model with pre-spawn starvation are presented in Fig S5 and S6.


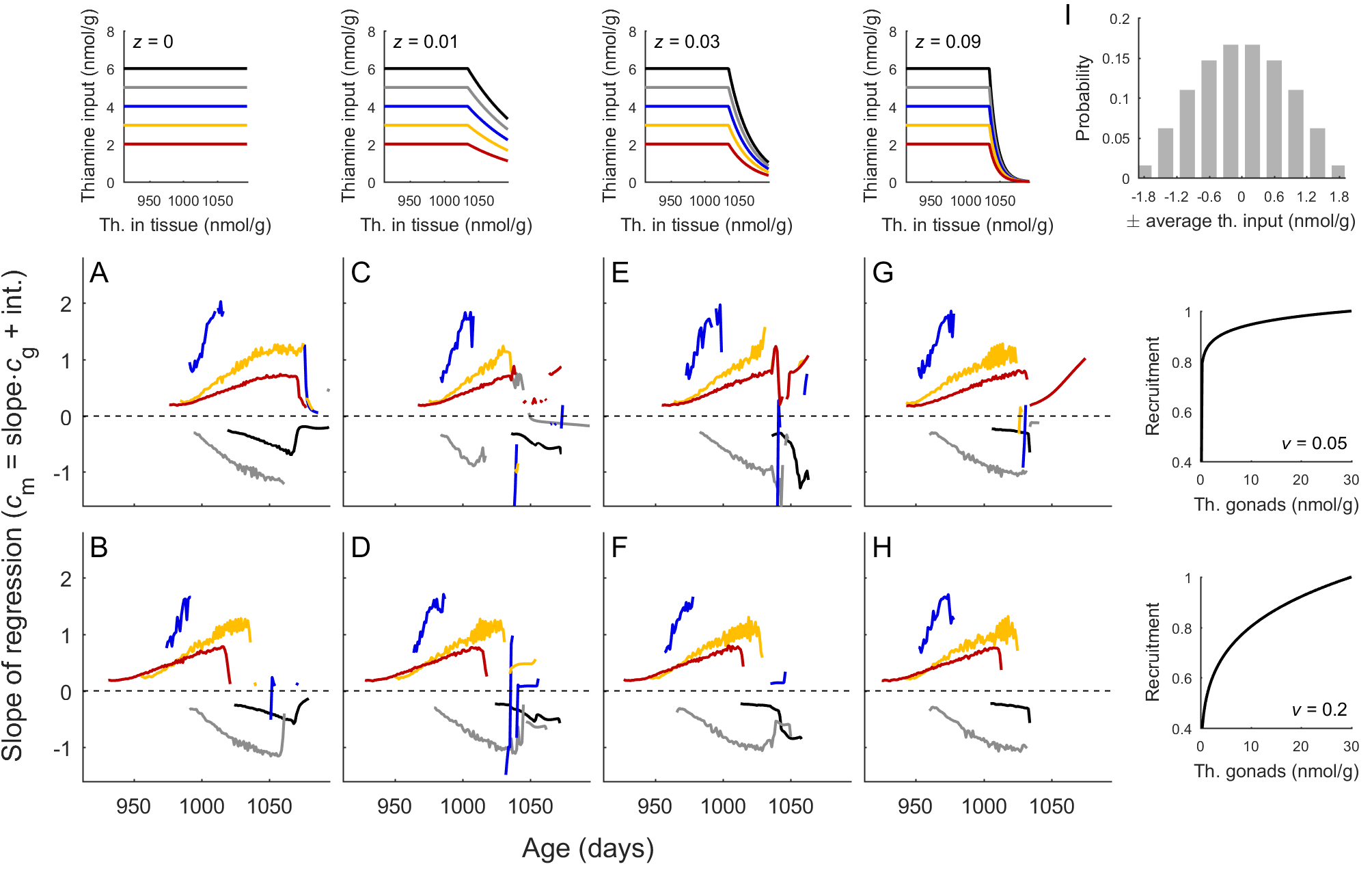

Fig S5. Correlation between gonad and muscle thiamine for scenarios with pre-spawn starvation. (A-H) Slopes of linear regression between thiamine concentration in gonads and muscles calculated for scenarios with different thiamine input levels *c*_b_ prior to starvation and the rate of the thiamine input decline during starvation (see panels in top row). Correlations are shown for different concentration-dependent scaling of juvenile recruitment (panels in utmost right column). Thin dashed black lines demarcate positive and negative slopes of the regression calculated for thiamine content in gonads (predictor variable) and muscles (response variable). The values of the exponents *z* and *v*, set the reduction of thiamine input level during starvation and concentration-dependent recruitment (see eq. S.1 and eq. 1 in the main text), are given in the panels. Scenarios were modelled with scaling of the concentration-dependent adult survival set by *u*=0.05 (cf. eq. 2 in the main text), and the rate of thiamine loss set by *b*=200 (see eq. 4 in the main text). Regressions slopes are plotted for cases with R^2^ greater than 0.2 and gonad thiamine variation greater than 0.5 nmol.


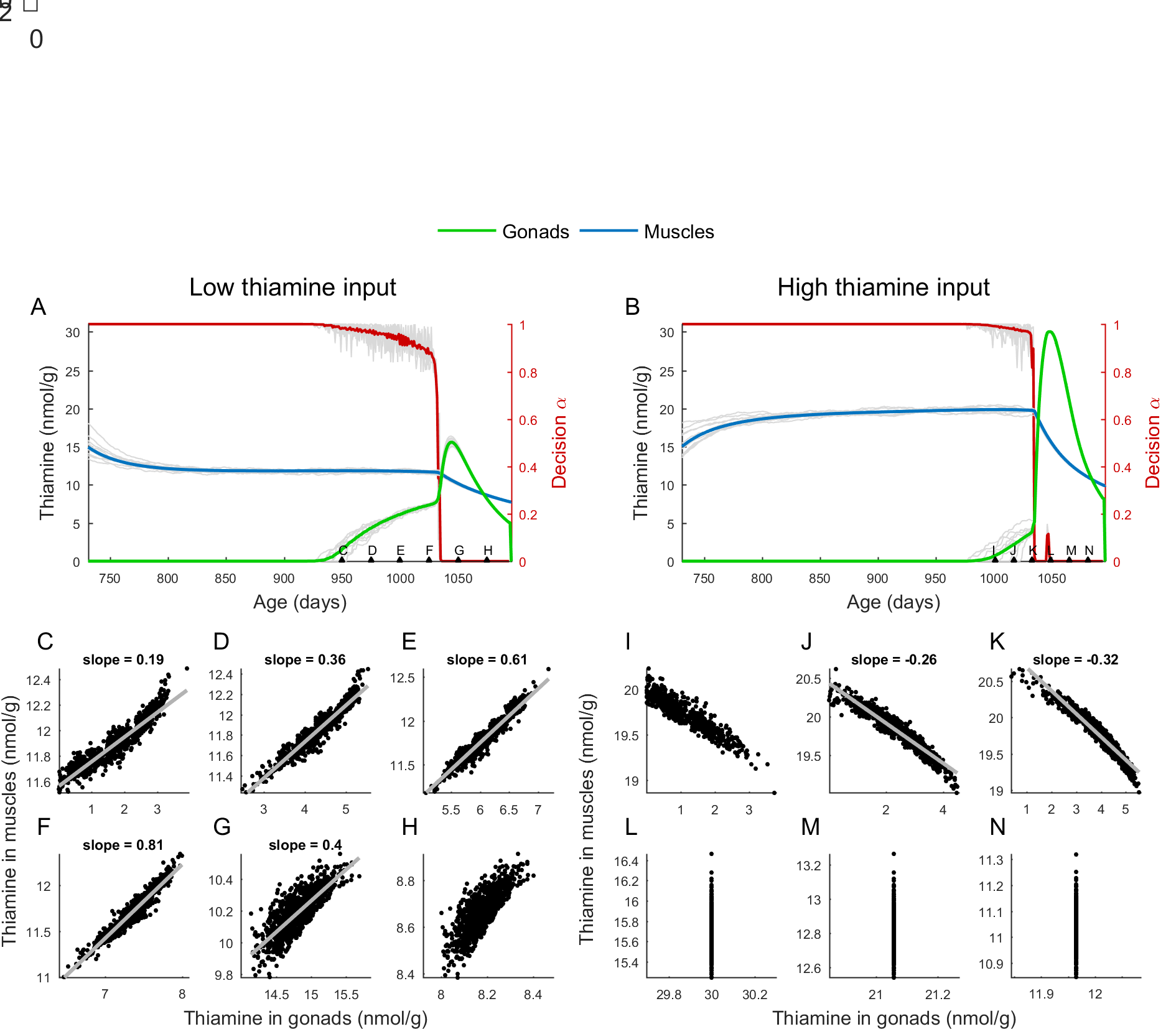


Fig S6. Thiamine concentration dynamics for scenarios with pre-spawn starvation*.* (A-B) The dynamics of thiamine level in gonads and muscles for scenarios of low and high thiamine input (set by *c*_b_ of 2 and 6 nmol/g respectively) and two months of pre-spawn starvation. Changes in thiamine tissue concentration are presented along with the proportion of thiamine allocated between muscles (*α*=1 corresponds to all thiamine converted to TDP in muscles) and gonads (*α*=0 corresponds to all thiamine allocated to gonads). Individual variation is represented by the light grey lines. Points labelled C-N match days for calculated linear regressions shown in panels C-N. (C-N) Linear regressions, with slopes given at the top of panels, for (C-H) low and (I-N) high thiamine input scenarios. Scenarios were modelled with the concentration-dependent probability of survival *p* shaped by the exponent *u*=0.05 (see eq. 2 in the main text), the probability of juvenile recruitment shaped by the *v*=0.05 (see eq. 1 in the main text), and rate of thiamine loss shaped by the exponent *b*=200 (see eq. 4 in the main text). The thiamine level-dependent survival of the post-spawning migration *p*_f_ was set by the exponent *l*=0.2 (see eq. 3 in the main text).

**6. *Tissue-specific thiamine loss rates and conversion efficiency of free thiamine to TDP***


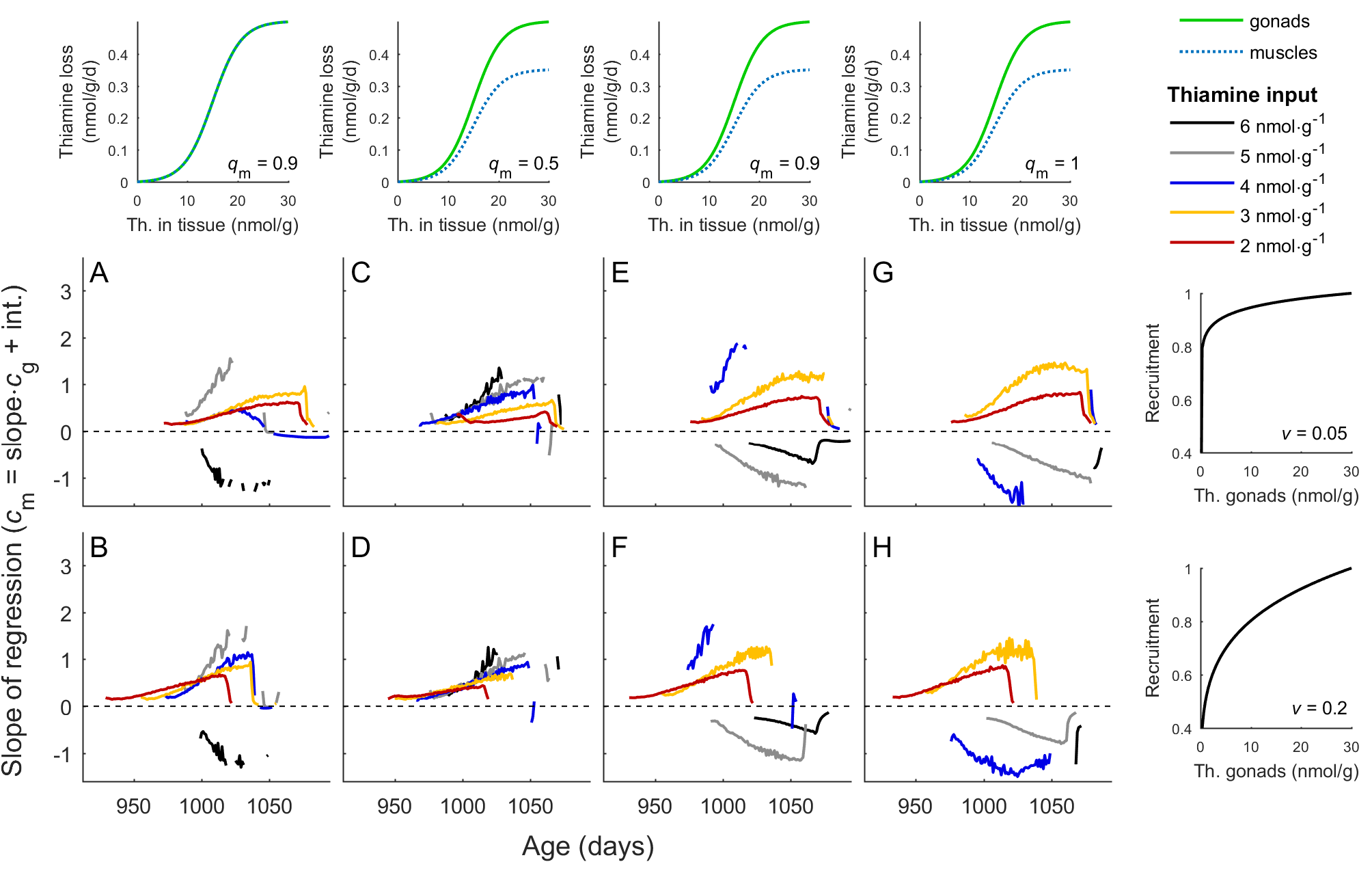

Fig S7. Correlation between gonad and muscle thiamine for different scenarios of thiamine loss and conversion efficiency of free thiamine to TDP. (A-H) Slopes of linear regression between thiamine concentration in gonads and muscles, modelled for different thiamine input levels *c*_b_ (see legend), and probability of juvenile recruitment (panels in rightmost column). The modelled scenarios differed in the tissue-specific rates of thiamine loss (see panels in the top row, and A-B vs. C-H) and in the values of *q*_m_, which determines the conversion efficiency of free thiamine to TDP (see *q*_m_ values in the panels in the top row). Thin dashed black lines demarcate positive and negative slopes of the regression calculated for thiamine content in gonads (predictor variable) and muscles (response variable). The values of the exponent *v* that scale the concentration-dependent recruitment (cf. eq. 1 in the main text), are given in the lower right corner of the panels in the rightmost column. Scenarios were modelled with scaling of the concentration-dependent probability of survival *p* shaped by the exponent *u*=0.05 (cf. eq. 2), and rate of thiamine loss shaped by the exponent *b*=200 (see eq. 4 in the main text). Regressions slopes are plotted for cases with R^2^ greater than 0.2 and gonad thiamine variation greater than 0.5 nmol.

**7. *Extended version of Fig 4 from the main manuscript***


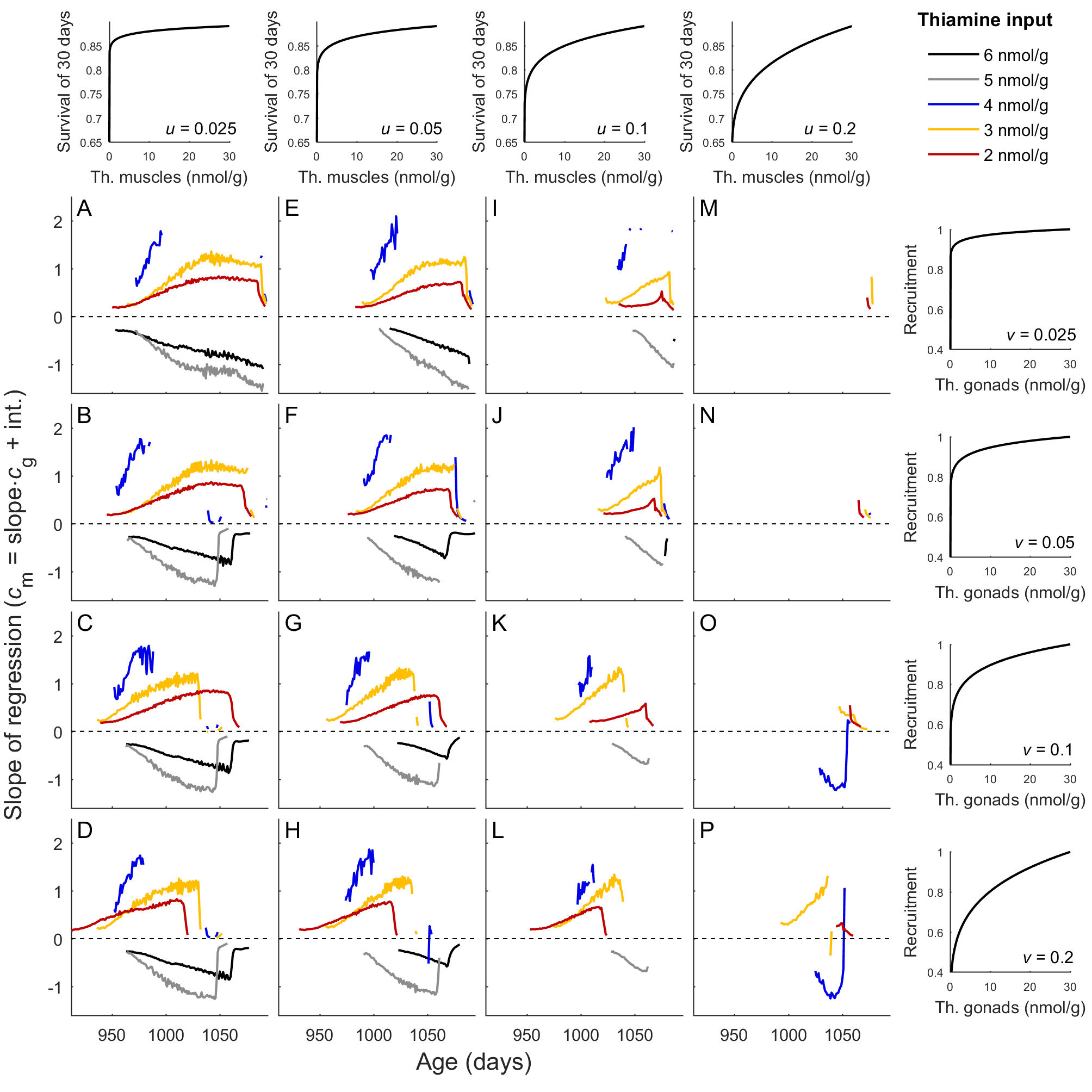

Fig S8. Correlation between gonad and muscle thiamine presented for different scenarios of concentration-dependent rates of adult survival and juvenile recruitment. (A-P) Slopes of linear regression between thiamine concentration in gonads and muscles calculated for scenarios with different thiamine input levels *c*_b_ (see legend), scaling of thiamine-dependent adult survival (panels in the top row), and probability of juvenile recruitment (panels in utmost right column). Thin dashed black lines demarcates positive and negative slopes of the regression calculated for thiamine content in gonads (predictor variable) and muscles (response variable). The values of the exponents *u* and *v*, which scale the concentration-dependent adult survival and recruitment (see eq. 1 and eq. 2 in the main text), are given in the top row and the utmost right column panels. Scenarios were modelled with scaling of the concentration-dependent rate of thiamine loss set by *b*=200 (see eq. 4 in the main text). The thiamine level-dependent survival of the post-spawning migration *p*_f_ was set by *l*=0.2 (see eq. 3 in the main text). Slopes of regressions calculated for a population of *N*=1000 females are presented for cases with R^2^ greater than 0.2 and gonad thiamine variation greater than 0.5 nmol.

**8. *Parameterisation of temporal changes in tissue mass***

Temporal changes in tissue mass in the model, including somatic body growth and seasonal changes in gonadal mass, were parameterized using data from growth curves, changes in blood mass and gonad somatic index in Atlantic salmon and other fish species (see below). Female somatic body mass in the model, consisting of muscle and blood, was given by *w*_s_=*w*_m_+*w*_b_, where *w*_m_ and *w*_s_ define the mass of muscles and blood (see Fig 1B). We parameterized the growth rate of somatic body by fitting a power function to the growth curve data for Atlantic salmon (Larsson 1984, Davidson et al. 2016) for the life stage prior to gonad filling (Naeve et al. 2018). Consequently, the growth of the somatic body *w*_s_ is given by
(S.2) *w*_s_(*t*) = 0.0013*t*^2.185^where *t* is the age of a female in days, and parameter values were obtained by fitting the function to the data with R^2^=0.95. We assumed that blood mass accounted for 6% of the somatic mass *w*_b_=0.06*w*_s_ (see (Garain and Mistry 2022)), and for the sake of simplicity the remainder was assumed to be muscles mass *w*_m_. Note, that the conclusions of our work did not change when the blood volume was assumed to be a few percentage points higher or lower as proportion of the somatic body, or scaled allometrically with body mass *w*_s_ (cf. (Garain and Mistry 2022)). Next, we used data on GSI from Nave et al. (2018) to parameterize seasonal changes of the mass of reproductive tissues *w*_g_. We assumed that minimum GSI in the model, including the time immediately after spawning is equal to 1. This means that the mass of gonads *w*_g_ was always greater or equal to 1% of somatic body mass *w*_s_. Gonad mass *w*_g_ during the period gonad filling from 1^st^ of April to 1^st^ of September (cf. (Naeve et al. 2018)), was given by an exponential function
(S.3) GSI(*u*) = *w*_s_0.966e^0.021^*^u^*
*u* defining the day starting 1^st^ of April (start of gonad filling) to 30^th^ of August (one day prior to spawning), and parameter values were obtained by fitting the function to the data with R^2^=0.82.

**9. *Simulations with low thiamine input and low excretion rates***

To keep our model simple, we did not explicitly consider the thiamine stored in the liver. In our main study, the absence of liver thiamine storage was compensated for by modelling thiamine input scenarios involving relatively high blood thiamine levels. However, simulations of low thiamine input and excretion rates showed that the conclusions of our study also apply to organisms for which liver storage plays a negligible role in thiamine balance (see Fig. S9).


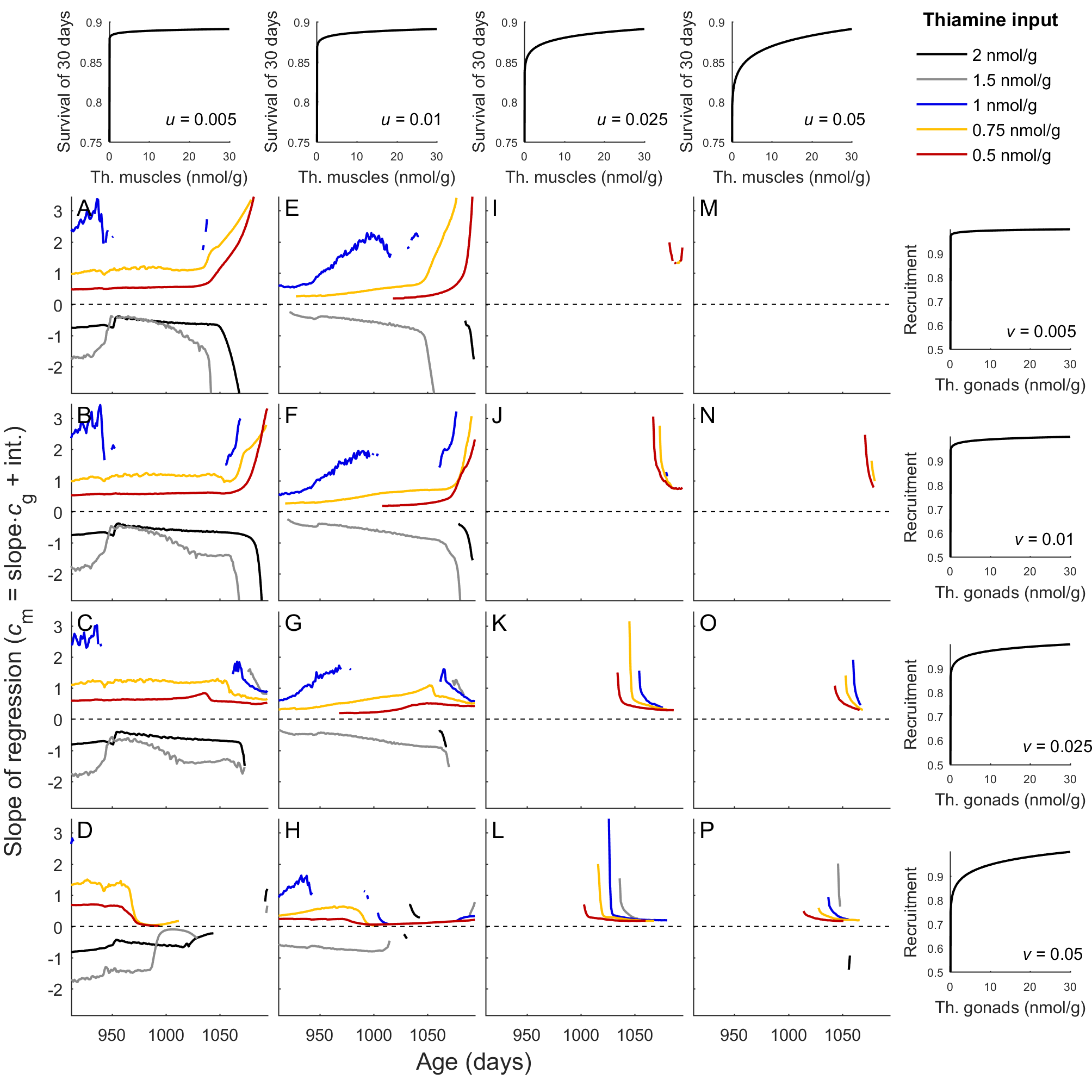

Fig S9. Correlation between gonad and muscle thiamine presented for low-level scenarios of thiamine input. (A-P) Slopes of linear regression between thiamine concentration in gonads and muscles calculated for scenarios with different thiamine input levels *c*_b_ (see legend), scaling of thiamine-dependent adult survival (panels in the top row), and probability of juvenile recruitment (panels in utmost right column). Thin dashed black lines demarcates positive and negative slopes of the regression calculated for thiamine content in gonads (predictor variable) and muscles (response variable). The values of the exponents *u* and *v*, which scale the concentration-dependent adult survival and recruitment (see eq. 1 and eq. 2 in the main text), are given in the top row and the utmost right column panels. Scenarios were modelled with scaling of the concentration-dependent rate of thiamine loss set by *b*=200 (see eq. 4 in the main text). The thiamine level-dependent survival of the post-spawning migration *p*_f_ was set by *l*=0.2 (see eq. 3 in the main text). The maximal mass-specific rate of thiamine excretion (nmol/g/d) *k*_x_, was set to 0.05 for gonads and 0.035 for muscles. Slopes of regressions calculated for a population of *N*=1000 females are presented for cases with R^2^ greater than 0.2 and gonad thiamine variation greater than 0.5 nmol.

**10. *References***

Davidson, J., T. May, C. Good, T. Waldrop, B. Kenney, B. F. Terjesen, and S. Summerfelt. 2016. Production of market-size North American strain Atlantic salmon Salmo salar in a land-based recirculation aquaculture system using freshwater. Aquacultural Engineering **74**:1-16.

Garain, A., and R. Mistry. 2022. Studies on blood volume in a teleostean fish, Mystus bleekeri in relation to body weight in summer and winter seasons. Flora and Fauna **28**:107-114.

Koski, P., C. Backman, and O. Pelkonen. 2005. Pharmacokinetics of thiamine in female Baltic salmon (Salmo salar L.) broodfish. Environ Toxicol Pharmacol **19**:139-152.

Larsson, P.-O. 1984. Growth of Baltic salmon Salmo salar in the sea. Marine Ecology Progress Series **17**:215-226.

Naeve, I., M. Mommens, A. Arukwe, and E. Kjørsvik. 2018. Ultrasound as a noninvasive tool for monitoring reproductive physiology in female Atlantic salmon (Salmo salar). Physiol Rep **6**:e13640.
